# Kazak mitochondrial genomes provide insights into the human population history of Central Eurasia

**DOI:** 10.1371/journal.pone.0277771

**Published:** 2022-11-29

**Authors:** Ayken Askapuli, Miguel Vilar, Humberto Garcia-Ortiz, Maxat Zhabagin, Zhaxylyk Sabitov, Ainur Akilzhanova, Erlan Ramanculov, Uli Schamiloglu, Angelica Martinez-Hernandez, Cecilia Contreras-Cubas, Francisco Barajas-Olmos, Theodore G. Schurr, Zhaxybay Zhumadilov, Marlen Flores-Huacuja, Lorena Orozco, John Hawks, Naruya Saitou

**Affiliations:** 1 School of Sciences and Humanities, Nazarbayev University, Astana, Kazakhstan; 2 National Center for Biotechnology, Astana, Kazakhstan; 3 National Laboratory Astana, Nazarbayev University, Astana, Kazakhstan; 4 Department of Integrative Biology, University of Wisconsin-Madison, Madison, Wisconsin, United States of America; 5 The Genographic Project, National Geographic Society, Washington, DC, United States of America; 6 Department of Anthropology, University of Maryland, College Park, Maryland, United States of America; 7 Immunogenomics and Metabolic Diseases Laboratory, National Institute of Genomic Medicine, Mexico City, Mexico; 8 L.N. Gumilyov Eurasian National University, Astana, Kazakhstan; 9 Department of Anthropology, University of Pennsylvania, Philadelphia, Pennsylvania, United States of America; 10 School of Medicine, Nazarbayev University, Astana, Kazakhstan; 11 Department of Anthropology, University of Wisconsin-Madison, Madison, Wisconsin, United States of America; 12 Population Genetics Laboratory, National Institute of Genetics, Mishima, Shizuoka, Japan; 13 Department of Biological Sciences, Graduate School of Science, University of Tokyo, Tokyo, Japan; 14 Advanced Medical Research Center, Faculty of Medicine, University of the Ryukyus, Okinawa Ken, Japan; Universitat Pompeu Fabra, SPAIN

## Abstract

As a historical nomadic group in Central Asia, Kazaks have mainly inhabited the steppe zone from the Altay Mountains in the East to the Caspian Sea in the West. Fine scale characterization of the genetic profile and population structure of Kazaks would be invaluable for understanding their population history and modeling prehistoric human expansions across the Eurasian steppes. With this mind, we characterized the maternal lineages of 200 Kazaks from Jetisuu at mitochondrial genome level. Our results reveal that Jetisuu Kazaks have unique mtDNA haplotypes including those belonging to the basal branches of both West Eurasian (R0, H, HV) and East Eurasian (A, B, C, D) lineages. The great diversity observed in their maternal lineages may reflect pivotal geographic location of Kazaks in Eurasia and implies a complex history for this population. Comparative analyses of mitochondrial genomes of human populations in Central Eurasia reveal a common maternal genetic ancestry for Turko-Mongolian speakers and their expansion being responsible for the presence of East Eurasian maternal lineages in Central Eurasia. Our analyses further indicate maternal genetic affinity between the Sherpas from the Tibetan Plateau with the Turko-Mongolian speakers.

## Introduction

Central Asia is a region that has witnessed important events in modern human population history, including the westward migration of nomadic pastoralists [1, 2], the eastward expansion of Indo-European speakers [3–5], and admixture between anatomically modern humans and archaic humans [6–9]. Notwithstanding the historical significance of the region, in-depth, comprehensive genetic studies of human populations in Central Asia are generally sparse. While the distribution of paternal lineages in Central Asia and Siberia is associated with language and ethnic identity [10, 11], maternal lineages do not exhibit any associations with language and ethnic group, except for an increase of West Eurasian mtDNA haplogroups in an east-to-west direction [12].

Previous studies have showed that human populations in Central Asia, including Kazaks, harbor elevated frequencies of both East and West Eurasian maternal lineages [12–14]. To date, there are several studies of maternal lineages in Kazak populations from the Altay Republic (Russia) [12, 15], from Xinjiang (China) [16, 17], and from Kazakhstan [13, 14, 18, 19]. While most of these studies sequenced the mitochondrial DNA (mtDNA) control region and genotyped coding region diagnostic SNPs, few studies analyzed the mitochondrial genomes (mtGenomes) of Kazaks (N = 19) [15, 20–22]. These studies again revealed the presence of both East and West Eurasian lineages in Kazak populations. Ingman and Gyllensten (2007) sequenced mitochondrial genomes (haplogroups A, D, and T) from six Kazaks. Derenko et al. (2012, 2014) presented whole mitochondrial genome sequences for twelve Kazaks, which belong to haplogroups M, H, HV, and U. Besides, Sahakyan and colleagues published a single Kazak mtGenome of haplogroup U in 2017 [21].

Historically speaking, there are three major divisions of Kazak populations. They include the Great Jüz, Middle Jüz, and Junior Jüz. The three Jüz occupy different, albeit overlapping, regions of Kazakhstan and bordering countries. While the Junior Jüz resides in the west, the Middle Jüz inhabits central, north, and east Kazakhstan, and the Great Jüz lives in southern regions of the country.

**Fig 1:**
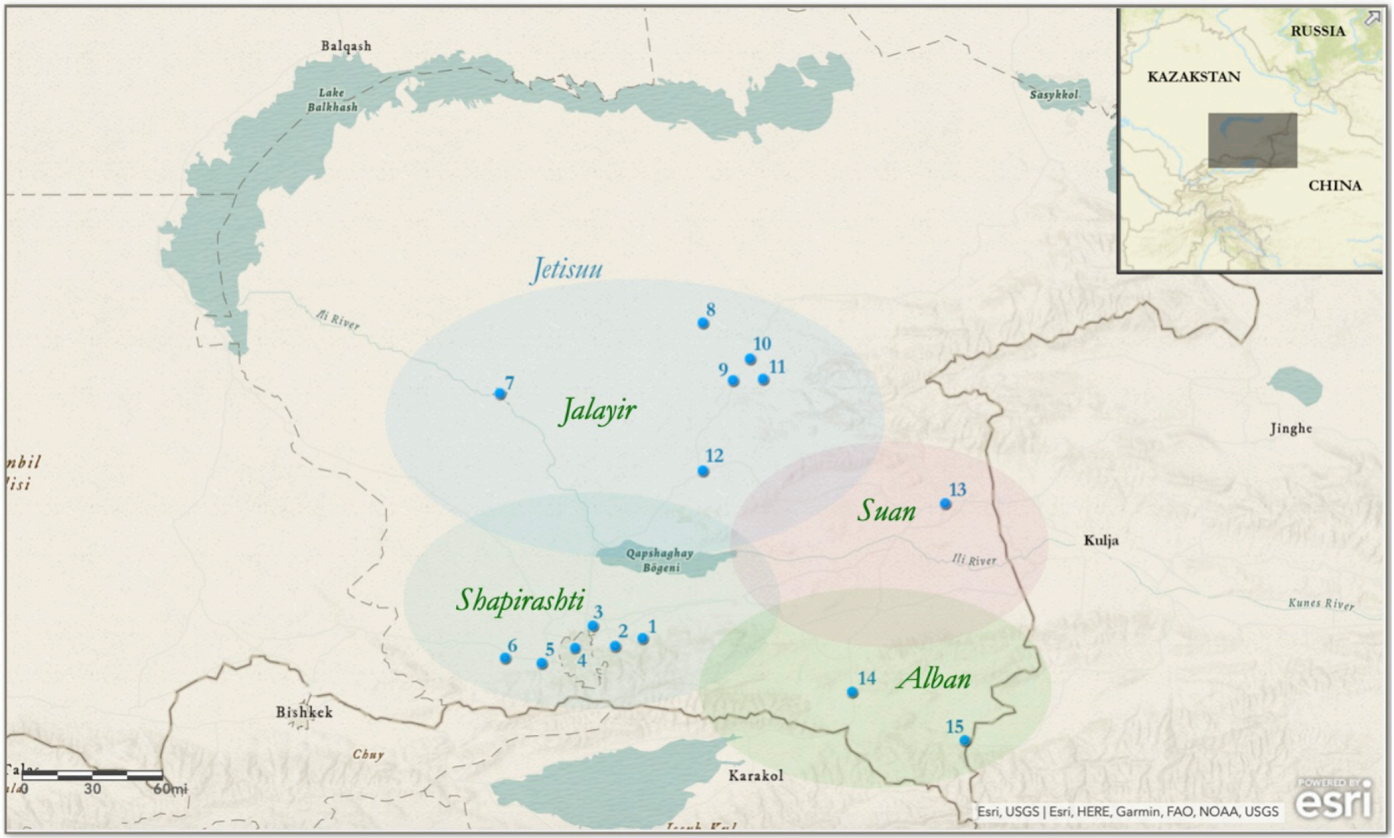
Map of Jetisuu and the sampling sites. 1. Engbekshi Kazak; 2. Talghar; 3. Ile; 4. Almati; 5. Karasay; 6. Jambil; 7. Balkash; 8. Karatal; 9. Köksuu; 10. Taldikorghan; 11. Eskeldi; 12. Kerbulak; 13. Panfilov; 14. Kegen; 15. Rayimbek.

To evaluate the maternal lineages of Kazaks at a finer scale, and to better understand the origins and dispersal of maternal lineages in Central Eurasia, we analyzed the mitochondrial genome data of 200 Kazak individuals, including full mtGenomes from 120 individuals and microarray data based pseudo-mtGenomes for 80 individuals, all living in the Jetisuu region of southern Kazakhstan (Fig 1).

These individuals belong to four clans of the Great Jüz, including Jalayir, Alban, Shapirashti, and Suan. Our results show that while a diverse array of haplogroups of Eastern and Western Eurasian origins comprise Kazak maternal lineages, Eastern Eurasian haplogroups, including A, B, C, D, F and G, account for about 50% of maternal genetic diversity of Jetisuu Kazaks. These findings indicate that mtDNA can help delineate historical processes of population formation in communities that are patrilineal in their social organization. We also performed comparative analyses of mtDNA variation in Jetisuu Kazaks and previously published mtGenomes (N = 2074) from 21 ethnic groups from across Central Eurasia. Our analyses indicate that mtDNA haplogroups are associated with language background at the language family level, while the presence of East Eurasian lineages in Central Eurasia is associated with the expansion of Turko-Mongolian speakers.

## Results

### mtDNA haplogroups in Jetisuu Kazaks

As shown in Table 1, Jetisuu Kazaks harbor at least 56 different haplogroups. Seven of them (C4, D4, F1, G2, H2, T2, and U5) occur at an average frequency of 3.5% or higher and appear in at least three of the clans surveyed in this study. Another six haplogroups (A5, B4, C5, D5, N9, and U2) appeared at a frequency of 2.0–3.0% and occurred in at least two of the clans. The remaining 43 haplogroups were represented by just one to three individuals (0.5–1.5%). Only B4, D4, G2, H2, and U5 mtDNAs were found in all four Jetisuu Kazak populations. In addition, haplogroup and genetic diversity was associated with the sample size in the Jetisuu Kazak populations (Tables 1 and 2), with Jalayirs (N = 76) having the highest number of haplogroups and Suans (N = 26) having the lowest haplogroup diversity (Tables 1 and 2).

**Table 1:** Mitochondrial DNA haplogroup frequencies in Kazak populations from Jetisuu, Kazakhstan.

Hg	Alban		Jalayir		Shapirashti		Suan		JetiKaz		Hg	Alban		Jalayir		Shapirashti		Suan		JetiKaz	
A	1	*2*.*86*	2	*2*.*63*	1	*1*.*69*			4	*2*	I1					1	*1*.*69*			1	*0*.*5*
A12			1	*1*.*32*	1	*1*.*69*			2	*1*	I2					1	*1*.*69*			1	*0*.*5*
A14					1	*1*.*69*			1	*0*.*5*	I4			1	*1*.*32*					1	*0*.*5*
A5	1	*2*.*86*	1	*1*.*32*					2	*1*	J1	1	*2*.*86*			1	*1*.*69*	1	*3*.*85*	3	*1*.*5*
B4	1	*2*.*86*	1	*1*.*32*	1	*1*.*69*	1	*3*.*85*	4	*2*	K1			1	*1*.*32*					1	*0*.*5*
B5					2	*3*.*39*	1	*3*.*85*	3	*1*.*5*	M10	1	*2*.*86*							1	*0*.*5*
C4	1	*2*.*86*	6	*7*.*89*	4	*6*.*78*			11	*5*.*5*	M5			1	*1*.*32*					1	*0*.*5*
C5					3	*5*.*08*	1	*3*.*85*	5	*2*.*5*	M65					1	*1*.*69*			1	*0*.*5*
C7					1	*1*.*69*			1	*0*.*5*	M7					1	*1*.*69*	1	*3*.*85*	2	*1*
D1			1	*1*.*32*					1	*0*.*5*	N1	1	*2*.*86*	1	*1*.*32*					2	*1*
D4	5	*14*.*29*	10	*13*.*16*	11	*18*.*64*	5	*19*.*23*	32	*16*	N9	2	*5*.*71*	1	*1*.*32*			1	*3*.*85*	5	*2*.*5*
D5			3	*3*.*95*	3	*5*.*08*			6	*3*	R0	1	*2*.*86*							1	*0*.*5*
F1			6	*7*.*89*	2	*3*.*39*	3	*11*.*54*	11	*5*.*5*	R1			1	*1*.*32*					1	*0*.*5*
F2							1	*3*.*85*	1	*0*.*5*	R2	1	*2*.*86*							1	*0*.*5*
G2	2	*5*.*71*	7	*9*.*21*	5	*8*.*47*	1	*3*.*85*	15	*7*.*5*	R9	1	*2*.*86*	1	*1*.*32*					2	*1*
G3			1	*1*.*32*					1	*0*.*5*	T1	1	*2*.*86*	1	*1*.*32*					2	*1*
H	4	*11*.*43*			1	*1*.*69*	1	*3*.*85*	6	*3*	T2			4	*5*.*26*	5	*8*.*47*	2	*7*.*69*	11	*5*.*5*
H1	1	*2*.*86*			2	*3*.*39*			3	*1*.*5*	U2	1	*2*.*86*	3	*3*.*95*					4	*2*
H10			1	*1*.*32*					1	*0*.*5*	U4	1	*2*.*86*	1	*1*.*32*			1	*3*.*85*	3	*1*.*5*
H13			2	*2*.*63*	1	*1*.*69*			3	*1*.*5*	U5	2	*5*.*71*	2	*2*.*63*	2	*3*.*39*	1	*3*.*85*	7	*3*.*5*
H2	2	*5*.*71*	8	*10*.*53*	3	*5*.*08*			13	*6*.*5*	U7	1	*2*.*86*			1	*1*.*69*			2	*1*
H3	1	*2*.*86*							1	*0*.*5*	V14							1	*3*.*85*	1	*0*.*5*
H44							1	*3*.*85*	1	*0*.*5*	W3			1	*1*.*32*					1	*0*.*5*
H5							1	*3*.*85*	1	*0*.*5*	W6			1	*1*.*32*					1	*0*.*5*
H6			1	*1*.*32*					1	*0*.*5*	X2i					1	*1*.*69*			1	*0*.*5*
H8			1	*1*.*32*	1	*1*.*69*			2	*1*	Z1							2	*7*.*69*	2	*1*
H9	1	*2*.*86*							1	*0*.*5*	Z3			1	*1*.*32*					1	*0*.*5*
HV					1	*1*.*69*			1	*0*.*5*	Z4			1	*1*.*32*					1	*0*.*5*
HV1					1	*1*.*69*			1	*0*.*5*											
HV14	1	*2*.*86*							1	*0*.*5*	***Hg***	24		33		26		18		59	
HV2			2	*2*.*63*					3	*1*.*5*	***N***	35		76		59		26		200*	

**Notes:** Haplogroups in this table are based on the haplogroup calls obtained via Haplogrep 2.0 [23] and mtPhyl 5.003 [24];

*The total number of mtDNA analyzed is ***N*** = 200, including four samples with ambiguous clan identity, but are from the four clans (their haplogroups are D4, HV2, C5, and N9, respectively) and 196 samples with unambiguous clan identities; Frequencies are shown in italics (%); **Hg:** Haplogroups; **N:** Number of samples; **JetiKaz**: all 200 Kazak samples from Jetisuu; Total number of haplogroups: 59.

**Table 2:** Results from Tajima’s Neutrality Test [33].

*Populations*	*Seq*. *Type*	*N*	*S*	*Ps*	*Θ*	*π*	*D*
**Alban**	Pseudo	15	160	0.0096566	0.0029698	0.0016957	-1.8932561
	Genuine	20	192	0.0115879	0.0032663	0.0018621	-1.7843685
	**Combined**	**35**	**308**	**0.0185889**	**0.0045138**	**0.0019007**	**-2.2005190**
**Jalayir**	Pseudo	25	208	0.0125536	0.0033246	0.0019530	-1.6451853
	Genuine	51	443	0.0267367	0.0059425	0.0021242	-2.3341813
	**Combined**	**76**	**526**	**0.0317460**	**0.0064770**	**0.0021572**	**-2.3233549**
**Shapirashti**	Pseudo	27	221	0.0133382	0.0034605	0.0019516	-1.7178648
	Genuine	32	276	0.0166576	0.0041362	0.0020340	-1.9549262
	**Combined**	**59**	**383**	**0.0231155**	**0.0049751**	**0.0021047**	**-2.0590438**
**Suan**	Pseudo	13	142	0.0085702	0.0027617	0.0018354	-1.5346953
	Genuine	13	153	0.0092341	0.0029757	0.0020451	-1.4321028
	**Combined**	**26**	**244**	**0.0147263**	**0.0038591**	**0.0020544**	**-1.8558244**
**Jetisuu Kaz**	Pseudo	80	412	0.0248657	0.0050204	0.0019058	-2.1460627
	Genuine	120	689	0.0415837	0.0077574	0.0020337	-2.4686793
	**Combined**	**200**	**854**	**0.0515420**	**0.0087761**	**0.0020823**	**-2.4548640**

**Notes:** This analysis involved 200 mitochondrial sequences. Codon positions included were 1st+2nd+3rd+Noncoding. All ambiguous positions were removed for each sequence pair (pairwise deletion option). There were 16569 positions in the final dataset. Evolutionary analyses were conducted in MEGA X [34].

**Abbreviations: *N*** = number of sequences, **S** = Number of segregating sites, ***Ps*** = S/n, **n** = total number of sites, **Θ** = Ps/a, **π** = nucleotide diversity, and ***D*** is the Tajima test statistic [35].

### mtDNA control region based PCA analysis

**Fig 2:**
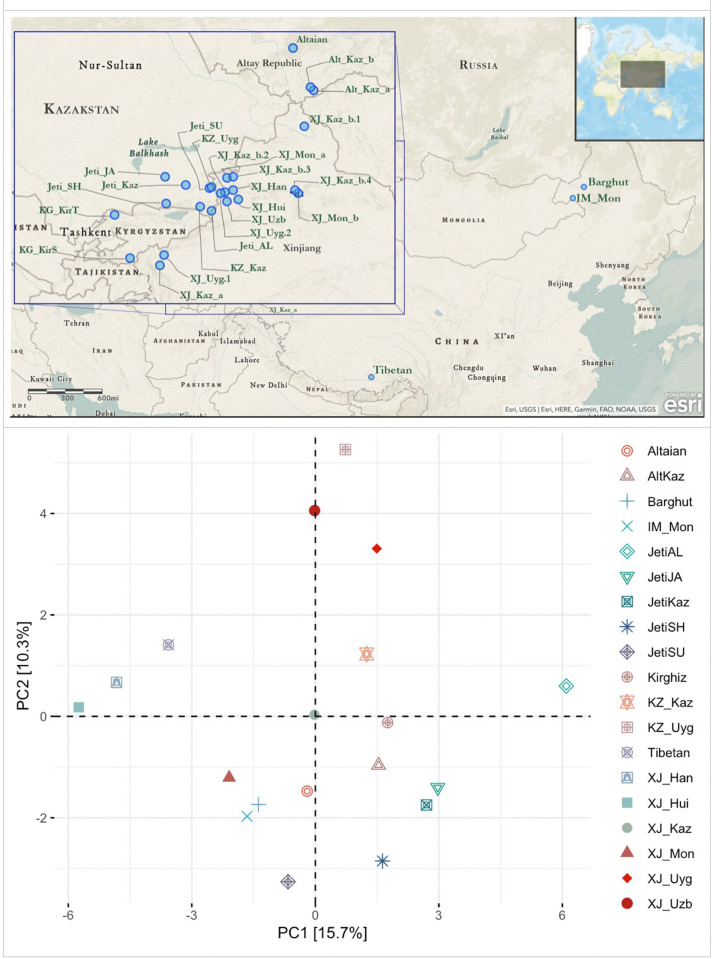
Geographic distribution of the human populations and the mtDNA control region haplogroup frequency based PCA. **Notes on Fig 2: Jeti_AL** [Alban (N = 35)], **Jeti_JA** [Jalayir (N = 76)], **Jeti_SH** [Shapirashti (N = 59)], and **Jeti_SU** [Suan (N = 26] are the four Kazak populations from Jetisuu, Kazakhstan; **Jeti_KZ** [Four populations from Jetisuu combined, plus 4 individuals with ambiguous clan identity (N = 200)]; **Altaian** [Altaians (N = 490) from the Altay Republic, Russia] [25]; **AltKaz_a** [Kazaks (N = 237) from the Altay Republic, Russia] [12]; **AltKaz_b** [Kazaks (N = 98) from the Altay Republic, Russia] [15]; **Barghut** [Barghuts (N = 149) from Hulunbuir, Inner Mongolia, China][15]; **IM_Mon** [Mongols (N = 48) from Inner Mongolia, China] [26]; **KZ_Uyg** [Uyghurs (N = 55) from Penjim, Panfilov, Almati, Kazakhstan]**, KG_KirT** [Kirghiz (N = 48) from Talas, Kirghizstan]**, KG_KirS** [Kirghiz (N = 47) from Sari-Tash, Kirghizstan]**, KZ_Kaz** [Kazaks (N = 55) from Kegen, Almati, Kazakhstan] [13]; **Tibetan** [Tibetans (N = 6109) from across Tibet, China] [27]; **XJ_Hui** [Dungans (Hui) (N = 45)], **XJ_Mon_a** [Mongols (N = 49)], **XJ_Kaz_a** [Kazaks (N = 53)], **XJ_Uzb** [Uzbeks (N = 58)], **XJ_Uyg** [Uyghurs (N = 47)], and **XJ_Han** [Han Chinese (N = 47)] [All six populations from Xinjinag, China] [17]; **XJ_Kaz_b** [Kazaks (N = 151) from several locations in Xinjiang, China, including b.1: Altay, b.2: Ile (Kulja), b.3: Buratala, and b.4: Urumqi, Sanji, and Kumul] [16]; **XJ_Mon_b** [Mongols (N = 106) from Xinjiang, China, but exact sampling site is unknown] [28].

We compared mtDNA profiles of the Jetisuu Kazaks with geographically adjacent populations based on haplogroup frequencies determined from the mtDNA control region sequences, since only control region data were available for most of the populations analyzed. As shown in the resulting PCA (Principal Component Analysis) plot, three groupings of populations appear in this analysis: (1) Turko-Mongolians clustered around indigenous Altaians; (2) Sino-Tibetan speaking groups; and (3) Uyghurs-Uzbeks (Fig 2). The Turko-Mongolian cluster was comprised of Turkic speaking Altaians at the center, surrounded by Turkic speakers (Kazak populations from Jetisuu (Kazakhstan), from Xinjiang (China), and from the Altay Republic (Russia), and Kirghiz from Kyrgyzstan) and Mongolic speakers (Mongols from Inner Mongolia and Xinjiang (China), and Barghuts from Inner Mongolia). Notwithstanding the vast geographic distance between them, Mongols and Barghuts from Inner Mongolia clustered close to Mongols, Kazaks, and Kirghiz in Central Asia. By contrast, Uyghurs and Uzbeks are genetically distant from their geographic, religious, and linguistic kin in Central Asia.

With regard to Kazak populations, they did not form a unique (single) cluster, but instead were scattered around the PCA plot (Fig 2). This distribution reveals an elevated maternal genetic diversity in Kazaks, as noted previously [12]. Mitochondrial haplogroup frequencies in Kazaks also varied depending on sampling sites and sample sizes. The most extreme disparities were observed between Kazak populations from Xinjiang (XJ_Kaz_a and XJ_Kaz_b), while an analogous situation was also observed in Altaian Kazaks (Alt_Kaz_a and Alt_Kaz_b), indicating the pronounced effect of sampling (size, coverage, location etc.).

**Fig 3:**
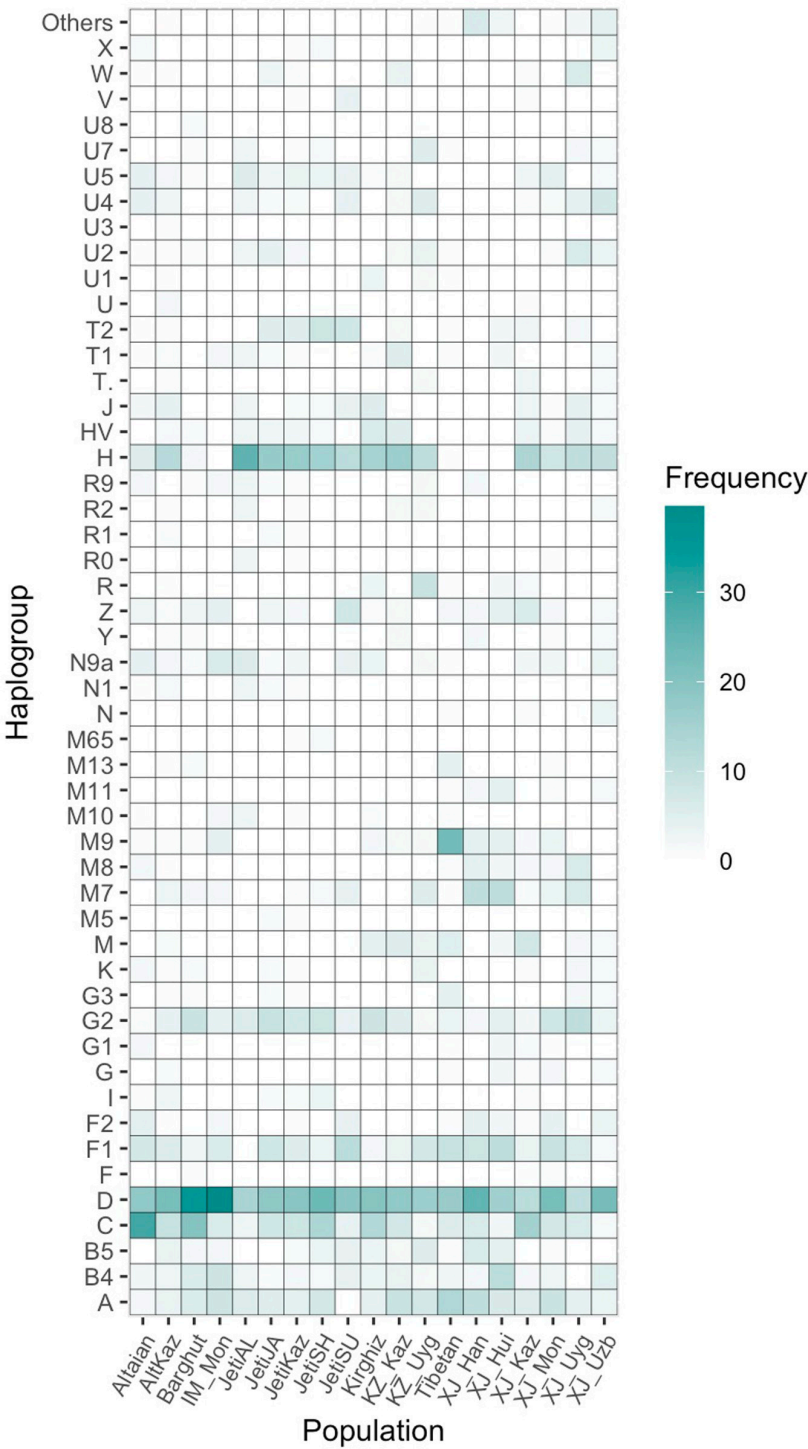
A heatmap for the mtDNA haplogroups and their relative frequencies. **Notes on Fig 3:** the heatmap was generated for the haplogroup frequency data that was used for PCA analysis in Fig 2.

Although living in proximity to each other, the four Kazak populations from Jetisuu exhibited considerable diversity in mtDNA lineages (Figs 2 and 3 and Table 1). While Albans had the highest number of West Eurasian lineages, Suans had the fewest. Jalayir and Shapirashti were positioned between Albans and Suans in the PCA plot. As indicated before, mtDNA haplogroup frequency distribution in Jetisuu Kazaks might have been influenced by sample size. Overall, no pronounced differences were observed in the phylogenetic affiliation of the four populations (SF-1).

As shown in Fig 3, East Eurasian haplogroups A, B, C, D, F, and G occurred in almost all populations in Central Asia, with D being the most frequent one. West Eurasian haplogroups (H, HV, V, J, T, U, W, and X) including several subbranches of R were also found in Turko-Mongolian speakers, albeit at relatively low frequencies. Haplogroups H and U were the most common West Eurasian haplogroup in Central Asia, and also occurred in the Jetisuu Kazaks at appreciable frequencies. In contrast, the West Eurasian haplogroups were almost absent in Sino-Tibetan speaking groups, but they harbored high frequencies of East Eurasian haplogroups M7 and M8.

In the PCA plot, Han Chinese and Dungan (Hui) from Xinjiang clustered together. Yet, the frequencies of the maternal lineages in Dungans considerably differed from those in Han Chinese. This result suggested that the maternal gene pool of Dungans did not exclusively derive from Han Chinese, even though Dungans supposedly shared a common maternal ancestry with them and speak Chinese. In Central Asia, intermarriages between Dungans and Turkic speakers are not uncommon because of shared religious beliefs. As an example, Dungans harbored West Eurasian maternal lineages such as U2, X2, T1, and HV [29], which are virtually absent in Han Chinese. The Han Chinese from Xinjiang also exhibited different maternal lineages compared to other Turko-Mongolian groups in Central Asia, indicating their recent arrival in Xinjiang. Despite the overall similarity of mtDNA diversity in Han Chinese and Tibetans, the latter clustered closer to Mongols and Kazaks.

Mongols from Xinjiang (XJ_Mon_a and XJ_Mon_b) and Inner Mongolia (IM_Mon) cluster together with Barghuts who speak a Mongolian language. Although characterized predominantly by East Eurasian maternal lineages, Mongolian speakers also harbored certain West Eurasian haplotypes (e.g., U, H, and HV, as shown in Fig 3) [15] at minimal frequencies, most likely due to their links with Turkic speakers in Central Asia. Xinjiang Mongols further shared multiple maternal lineages with Kazaks from Xinjiang and Kazakhstan. This finding might be explained by their common Altaic ancestry, geographic proximity, similarity in subsistence patterns, and intermarriage during the periods of Jungar, Kalmak, and Kazak Khanates.

By contrast, Mongols from Inner Mongolia shared a certain number of maternal haplotypes with Kazaks. This finding may imply their descent from a common ancestral group at the time of Genghis Khan or even before. Among Turkic speakers from Central Asia, Uyghurs and Uzbeks exhibited elevated frequencies of West Eurasian lineages, indicating that a substantial number of maternal lineages may have been contributed by Indo-European speaking peoples to the gene pools of Uyghurs and Uzbeks, even though they are Turkic speakers.

### Analysis of mtDNA variation

We subjected the mtDNA sequences to statistical analysis to evaluate patterns of diversity in Kazaks. Values of Tajima’s D are significantly deviated from neutrality, implying that population expansion and/or natural selection might have played a role in shaping genetic diversity in Kazaks (Table 2). The disparity between the number of segregating sites and the average number of nucleotide differences was more pronounced when the number of sequences was higher. However, it seems that negative Tajima’s D characterizes mitochondrial sequences (both control region and whole genome sequences) from many human populations [12, 30–32].

### Comparative phylogenetic analysis of mtGenomes from Central Eurasia

Here we discuss the genetic features of maternal lineages present in Kazaks from Jetisuu in the context of central Eurasia, including Central Asia, Siberia, and Eastern Europe. We retrieved over 2000 full mtGenome sequences from GenBank [36], and used them to conduct a comparative analysis of the maternal genetic diversity in the four language families in Eurasia, namely, Altaic, Uralic, Sino-Tibetan, and Indo-European (Table 3).

**Table 3:** Information on whole mitochondrial genome sequences which used for comparison.

Nr.	Language		Ethnicity	N	Reference
1	Altaic	Turkic	Altaian	35	Derenko et al. 2012; Derenko et al. 2010; Derenko et al. 2007;
2			AltKaz	11	Derenko et al. 2014;
			Kazak	7	Sahakyan et al. 2017; Ingman and Gyllensten 2007;
3			Kirghiz	125	Peng et al. 2018
4			Tatar	73	Malyarchuk et al. 2010
5			Tuba	33	Sukernik et al. 2012; Zhao et al. 2009; Volodko et al. 2008;
6			Uyghur	734	Peng et al. 2018; Zheng et al. 2017 [43]; Zhao et al. 2009
7			Uzbek	5	Sahakyan et al. 2017; Zhao et al. 2009; Ingman et al. 2000;
8		Mongolic	Barghut	14	Derenko et al. 2014; Derenko et al. 2012;
9			Buryat	231	Derenko et al. 2007; Derenko et al. 2010; Derenko et al. 2012; Derenko et al. 2014; Derenko et al. 2017/2018;
10			Kalmak	3	Palanichamy et al. 2015 [44]; Derenko et al. 2014; Derenko et al. 2010;
11			Mongol	7	Ingman & Gyllensten 2007; Sahakyan et al. 2017; Kong et al. 2003; Protasova et al. 2016 [45];
12	Uralic	Ugric	Hungarian	95	Malyarchuk et al. 2018
13	Sino-Tibetan	Tibetic	Tibetan	231	Zhao et al. 2009
14			Sherpa	76	Kang et al. 2013
15	Indo-European	Persian	Tajik	223	Peng et al. 2018
16		Slavic	Belrussian	7	Mielnik-Sikorska et al. 2013; Malyarchuk et al. 2008;
17			Czech	13	Mielnik-Sikorska et al. 2013; Malyarchuk et al. 2008;
18			Polish	30	Mielnik-Sikorska et al. 2013; Malyarchuk et al. 2008;
19			Slovak	22	Mielnik-Sikorska et al. 2013; Malyarchuk et al. 2008;
20			Russian	98	Mielnik-Sikorska et al. 2013; Malyarchuk et al. 2008; FTDNA;
21			Ukranian	1	Malyarchuk et al. 2008
Total number of mtG sequences (N):				2074	

Altaic speakers were the main focus of the analyses in the current study, since we aimed to understand their origins from the maternal perspective and evaluate the maternal gene pool of Kazaks in relation to those of other Altaic speakers. The mtDNA sequences of Slavs [37–39] and Tajiks [32] were included in the analysis in an effort to trace the origins of the West Eurasian maternal lineages (e.g., H, V, and U) found in Kazaks. We further included Hungarian sequences [40] to investigate whether Hungarians had any maternal genetic affinity with Kazaks and other Altaic speakers, since Hungarians have historical connections with Central Asia. Lastly, we analyzed mtGenomes of Tibetans [41] and Sherpas [42], because Tibetan speakers have been neighbors of Altaic speakers for millennia and have interacted with them during various time periods of history.

In the current study, we were able to build high resolution of phylogenetic trees from the mtGenome sequences of the Jetisuu Kazak populations and evaluate their maternal genetic affinities with populations from Central Asia and other regions. Comparative analyses were carried out using 2074 full mtGenome sequences from 21 ethnic groups (Tables 3 and S4). The analyses were performed in three rounds: (1) all sequences (N = 2274), including those from Jetisuu Kazaks and other Central Eurasian populations, were analyzed together to identify their affinities and haplogroup affiliations; (2) Central Eurasian and Jetisuu Kazak sequences that belonged to the haplogroups A, B, C, D, F, G, H, T, and U, were selected and aligned together with sequences from the Phylotree Build 17.0 [46] (N = 1155; Tables 4 and S5 and S6). Subsequent analyses were separately performed for each of the haplogroups. Below, we present the results of the final round of phylogenetic analyses, focusing on each of the major haplogroups which characterize maternal genetic composition of the Jetisuu Kazaks.

**Table 4:** Central Eurasian mtGenomes analyzed together with phylotree mtGenomes.

Count	Haplogroup	Jetisuu	Central Eurasia	Phylotree	Total N
1	**A**	9	53	26	**88**
2	**B**	4	13	7	**24**
3	**C**	17	68	22	**107**
4	**D**	37	112	57	**206**
5	**F**	12	42	22	**76**
6	**G**	17	54	28	**99**
7	**H**	28	126	116	**270**
8	**T**	13	60	38	**111**
9	**U**	15	123	36	**174**

**Fig 4:**
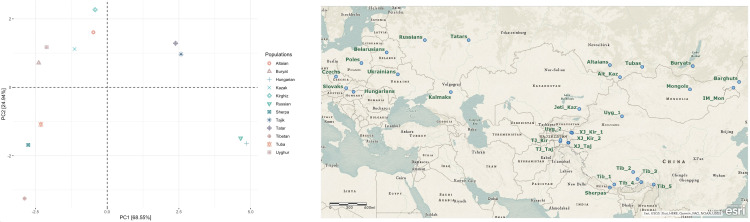
Geographic distribution of the human populations included in the analysis of mitochondrial genomes from Central Eurasia and the mtGenome pairwise F_ST_ value based PCA. **Notes on Fig 4:** Populations having more than 30 sequences were included in the PCA analysis, and thus the dataset contained 1954 sequences from previous studies (Table 3) and 120 Kazak mtGenome sequences from Jetisuu. Sample IDs of the sequences were given in S7 Table. The pseudo-mtGenomes (N = 80) were excluded from the PCA analysis. **Kazak** [Kazaks from Jetisuu, Kazakhstan, i.e., **Jeti_Kaz on the map**]; **Alt_Kaz** [Kazaks from the Altay Republic, Russia]; **Buryat** [Buryats from the Buryat Republic, Russia]; **Hungarian** [Hungarians from Hungary]; **Kirghiz** [Kirghiz from Xinjiang, China (**TJ_Kir**) and Kirghiz from Tajikistan (**TJ_Kir**)]; **Russian** [Russians from Russia]; **Sherpa** [Sherpas from Zhangmu, Tibet, China]; **Tajik** [Tajiks from Xinjiang, China (**XJ_Taj**) and Tajiks from Tajikistan (**TJ_Taj**)]; **Tatar** [Tatars from the Tatar Republic, Russia]; **Tibetan** [Ethnic Tibetans and other ethnic groups from Tibet, China (**Tib_1–5**)]; **Tuba** [Tubas from the Tuba/Tuva Republic, Russia]; **Uyghur** [Uyghurs from Xinjiang, China (Uyg**_1–2**)].

In order to evaluate genetic distance among populations, we estimated pairwise F_ST_ values and performed PCA analysis with them (Fig 4). In the PCA plot shown in Fig 4, Kirghiz, Hungarians, and Tibetans were positioned at the tips of a triangular pattern, indicating they were genetically quite distant from each other, while other populations were scattered largely around the edges of the imaginary triangle. Altaic speakers clustered together in the top left quarter of the PCA plot (Fig 4), with Tubas and Tatars being exceptions. While Tubas were positioned close to Sherpas, Tatars separated from the other Turkic speaking populations and clustered together with Tajiks. An unexpected observation was the separation of Sherpas from Tibetans, with the former being closer to Turkic speakers, especially Tubas. Interestingly, Hungarians were clustered together with Russians in the bottom right quarter of the PCA plot.

### Major East Eurasian Lineages

#### Haplogroup A

Haplogroup A is one of the founding haplogroups for Asia and the Americas. Haplogroup A was first identified by Schurr et al. [47] and further described by Torroni et al. [48] as one of the major maternal lineages of Native Americans. In our sample set from Jetisuu, we observed three major subbranches of A, namely A5, A12, and A14. The diverse branching pattern of haplogroup A suggested that it was one of the founding maternal lineages of Kazaks.

**Fig 5:**
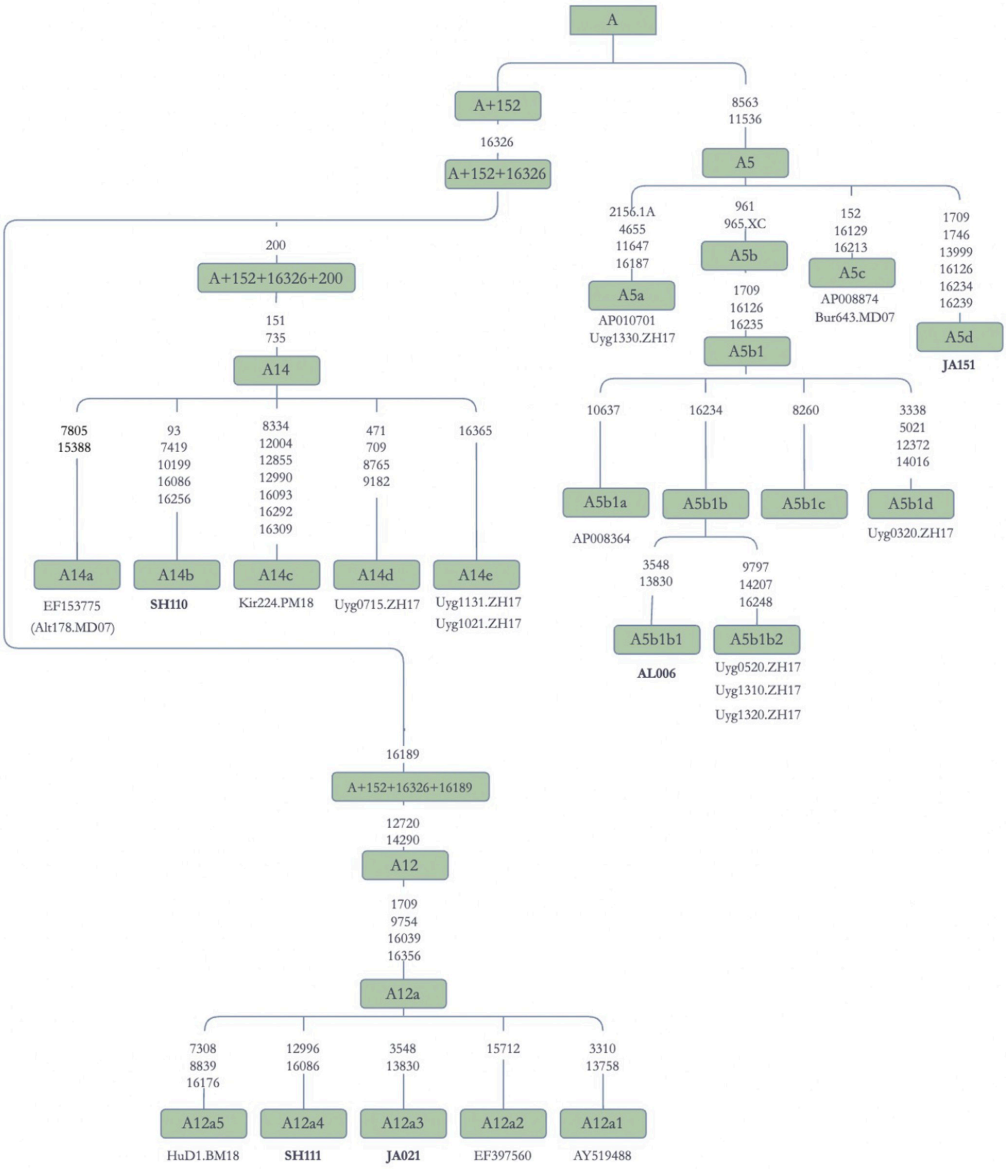
Schematic phylogenetic tree for the haplogroup A mitochondrial genomes. **Note:** Alt: Altaian; Uyg: Uyghur; Kir: Kirghiz; Hu: Hungarian.

Haplogroup A5 contained mtDNA sequences from Buryats, Kazaks, Uyghurs, and Japanese (Fig 5), with the Japanese sequences AP010701 and AP008874 being identical to those in Uyghurs and Buryats. This result suggested that Japanese have some maternal genetic affinities with Turkic speakers (Kazaks and Uyghurs) and Mongolic speakers (Buryats), as suggested by some models of Altaic language family evolution, e.g., Trans-Eurasian language model [49]. Kazaks, Uyghurs, and Japanese had additional mtDNA lineages representing multiple branches of A5, implying that the possibility of recent admixture was highly unlikely.

In the Phylotree Build 17.0 [46], A14 does not have any sub-branches. It contains only a single Altaian sequence (EF153775), which is included in our dataset (Alt178.MD07). Our phylogenetic analysis identified new subbranches of A14, which were formed by sequences from Turkic speaking Altaians (A14a), Kazaks (A14b), Kirghiz (A14c), and Uyghurs (A14d and A14e). This configuration suggested that these sequences might derive from a common Turkic maternal ancestor.

Two sequences from Jetisuu, along with a sister branch from Hungary, formed a distinct clade (A12a). The two Kazak sequences (JA021 and SH111) were named A12a3 and A12a4, respectively, whereas the Hungarian sequence (HuD1.BM18) was named A12a5. One of the two sequences representing A12a in the Phylotree Build 17.0 [46] came from an Evenk (EF397560), and the second from a Mansi (AY519488).

#### Haplogroup B

Haplogroup B is ubiquitous in Asia, Oceania, and Americas [12, 15, 25, 50–53]. It may have originated in Southeast Asia [50, 51], and is estimated to be 50 kyr old [54]. In support of this view, an mtDNA with the basal sequence motive of haplogroup B was found in a 40-kyr-old ancient human remain excavated from Tianyuan Cave near Beijing, China [55].

**Fig 6:**
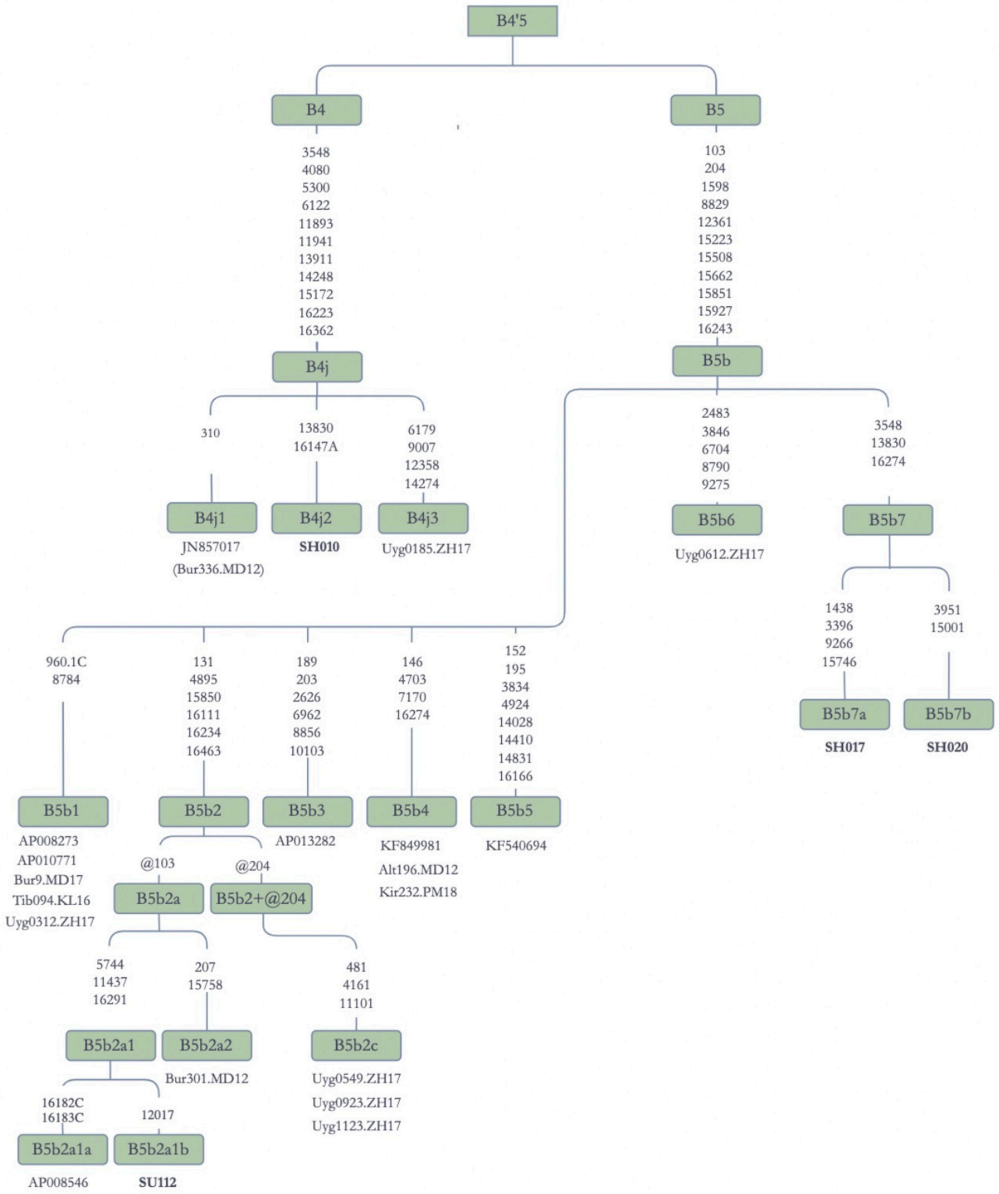
Schematic phylogenetic tree for the haplogroup B mitochondrial genomes. **Note:** Alt: Altaian; Bur: Buryat; Uyg: Uyghur; Kir: Kirghiz; Tib: Tibetan.

Two of its sub-branches, B4 and B5, were found in Jetisuu Kazak populations. In Fig 6, B4j contains three sequences, one each from a Kazak (B4j1), a Uyghur (B4j3), and a Buryat (B4j2), indicating that the haplogroup is shared among Turko-Mongolian speakers. With these data, we confirm the motif of B4j [*3548 4080 5300 6122 11893 11941 13911 14248 15172 16223 16362*] that was given a preliminary status in the Phylotree Build 17.0 [46].

A subclade of B4, B4b1a3, which was first described and named by Derenko and colleagues [15], was found in Altaians, Buryats, and Uyghurs (S4 Table). Found in Turko-Mongolian speakers, this haplotype is phylogenetically closest to the Native American B2 branch. B4c1b2b, a rare branch of B4 previously found in Chuvash people [15], has its sister branch (B4c1b2a) in Uyghurs (S4 Table), indicating the possible existence of the lineage in the Proto-Turkic stock population.

In our sample set, B5b was represented predominantly by sequences from Turko-Mongolian speakers (N = 13), including Turkic speaking Altaians, Kazaks, Kirghiz, and Uyghurs, and Mongolic speaking Buryats, except for a single sequence in a Tibetan. Two Kazak and one Uyghur sequences also did not belong to any previously known branches in the clade of B5b. Therefore, the Kazak sequences, SH017 and SH020, were named B5b7a and B5b7b, respectively, and the Uyghur sequence was named B5b6.

In the Phylotree Build 17.0 [46], we noted Japanese sequences which represented several sub-branches of B5b. For instance, Jetisuu Kazak sequence SU112 showed similarities with Japanese sequence AP008546 as shown in Fig 6. Although it is evident that Japanese mtDNAs show similarities to those in Turko-Mongolian speakers from Central Asia and Siberia, B5b seems to have a wide geographic distribution. B5b5 [KF540694] comes from Hakka, a Sinitic speaking native group from Taiwan [56], while B5b4 [KF849981] is (mostly likely) from Han Chinese [57].

#### Haplogroup C

Haplogroup C mtDNAs are found in human populations throughout Asia and the Americas [12, 15, 25, 51, 58]. In Jetisuu Kazak populations, seventeen individuals harbored haplogroup C mtDNAs, which represented 8.5% of the total sample size (Table 1). Except for C1, which is an American branch [59], all major sub-branches of haplogroup C appeared in the Jetisuu Kazak populations, including C4, C5, and C7. Most of the Jetisuu Kazak C4 sequences belonged to C4a, with one C4b and one C4d mtDNA also being present. A refined nomenclature was used for the subbranches of C4 after constructing a phylogenetic network for the mtGenome sequences.

**Fig 7:**
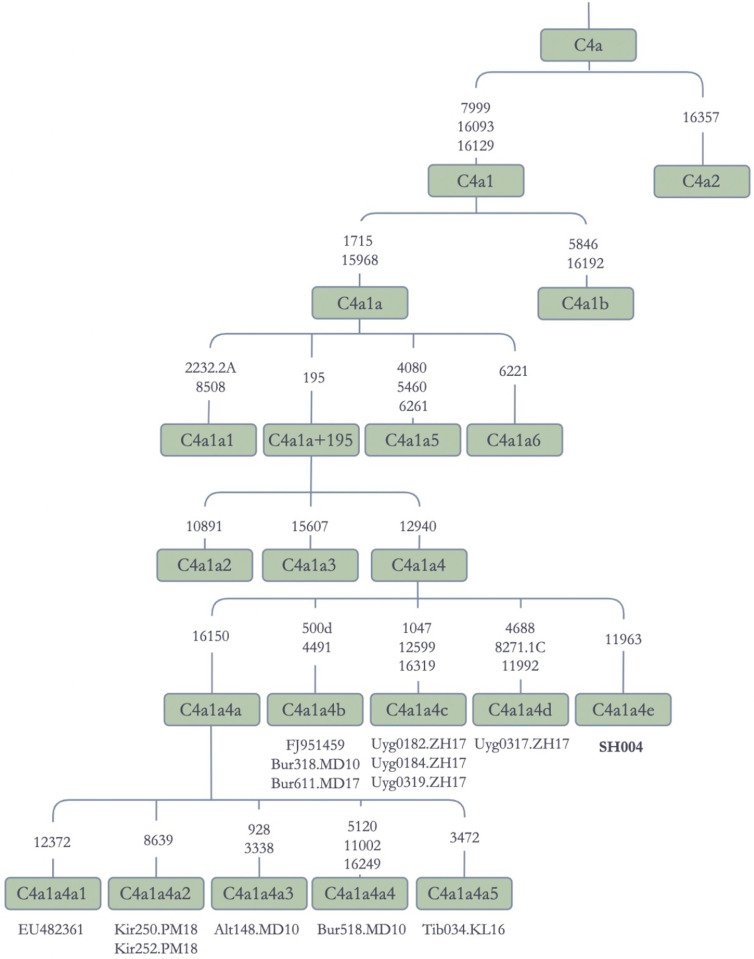
Schematic phylogenetic tree for the haplogroup C4a1 mitochondrial genomes. **Note:** Alt: Altaian; Bur: Buryat; Uyg: Uyghur; Kir: Kirghiz; Tib: Tibetan.

C4a1a encompassed sequences from mainly Turko-Mongolian speakers, but also contained sequences from Indo-European speakers, such as Russians (Rus32), Poles (Pol422), and Tajiks (Taj126 and others), Uralic speaking Hungarians (HuS22), and Sino-Tibetan speakers (Tib034), indicating much deeper temporal origin and wide dispersal of the clade (S4 Table and Fig 7). Similarly, C4a1a4 contained four Jetisuu Kazak sequences in addition to multiple sequences from various ethnic groups, including Altaians, Buryats, Kirghiz, Tibetans, and Uyghurs. The five sub-branches of C4a1a4 were named as C4a1a4a through C4a1a4e, of which C4a1a4e was represented by a Jetisuu Kazak sequence (Fig 7). The tree topology suggested that C4a1a4 could also be one of the founding maternal lineages for Turko-Mongolians. In the Phylotree Build 17.0 [46], C4a1a4 is represented by just two sequences from Siberia, one from the Shors and another from the Buryats, the latter being included in our dataset.

**Fig 8:**
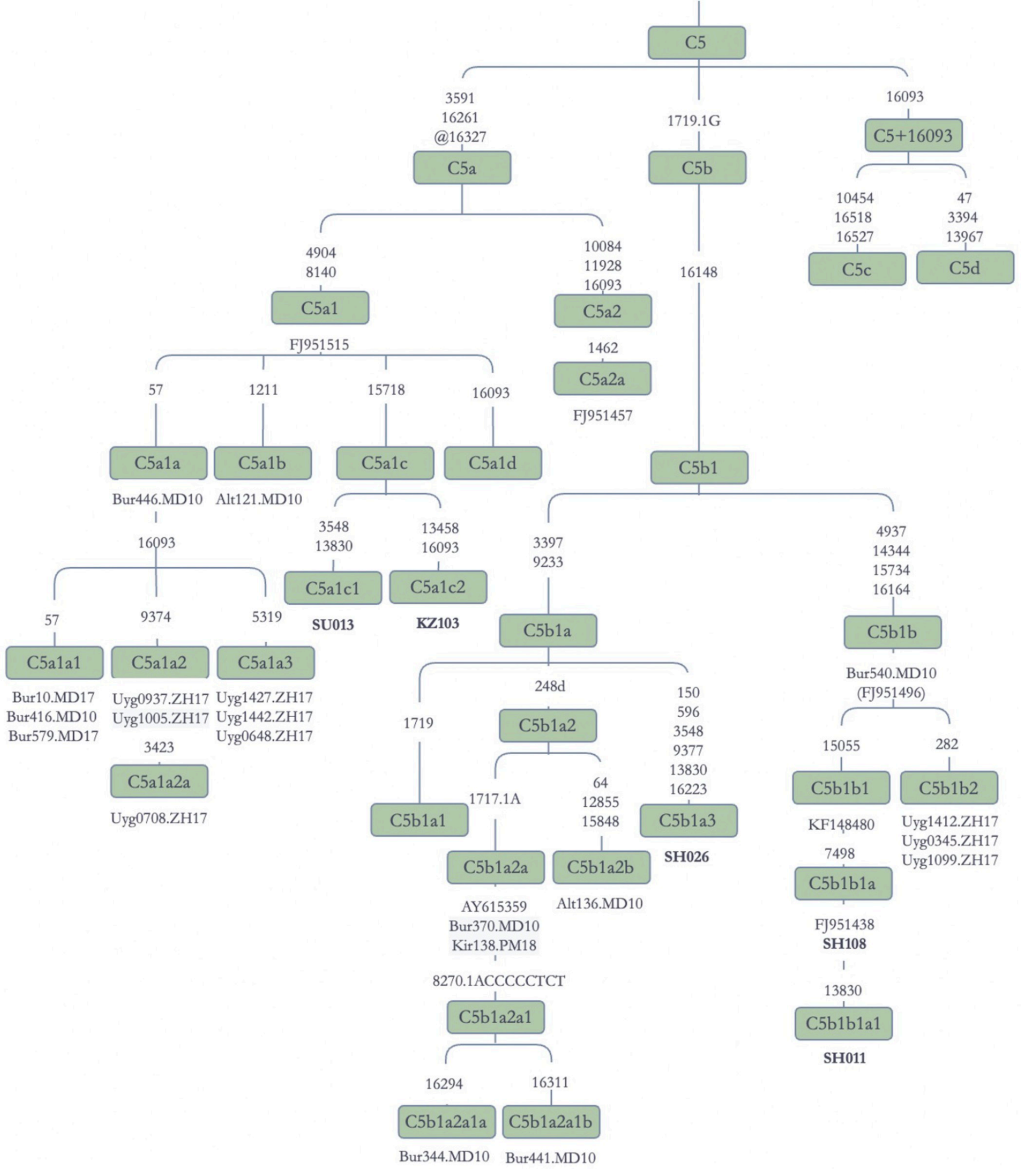
Schematic phylogenetic tree for the haplogroup C5 mitochondrial genomes. **Note:** Alt: Altaian; Bur: Buryat; Uyg: Uyghur; Kir: Kirghiz.

Haplogroup C5 has four sub-branches, including C5a, C5b, C5c, and C5d, each of which also having their own subclades. There are both C5a and C5b sequences in our Jetisuu Kazak populations (Fig 8). C5a1 is represented by a single branch in the Phylotree Build 17.0 [46]. Two of our Kazak sequences, SU013 and KZ103, clustered within this clade, and were named C5a1c1 and C5a1c2, respectively. Altaian, Buryat, and Uyghur sequences also grouped within this cluster, with the Altaian sequence (Alt121) being defined as C5a1b in the Phylotree Build 17.0 [46].

C5b1 was represented by sequences from Kazaks, Uyghurs, Buryats, and Yakuts (Fig 8). C5b1a included one Kazak sequence (SH026), and several other sequences from Altaians, Buryats, and Kirghiz, while Kazak sequence (SH026) belonged to C5b1a3. Two Jetisuu Kazak sequences, SH108 and SH011, belonged to subclade C5b1b1, which has a single branch represented by two Yakut sequences (FJ951438 and KF148480) in the Phylotree Build 17.0 [46]. Its sister branch C5b1b2 appeared in Uyghurs. The Kazak and Uyghur sequences were more derived compared to the ones found in Yakuts and Buryats, as shown in Fig 8. The diagnostic mutation for C5b, an insertion at np 1719 (1719.1G), should be reconsidered, since all 15 mtGenomes in our data set possessed a back mutation at this position (@1719.1G).

In light of this evidence, haplogroup C5 can be considered as one of the founder lineages of Turko-Mongolian speakers, or Altaic speakers more generally. All but one of the sequences in C5b belong to Turko-Mongolian speakers, including Altaians, Buryats, Kazaks, Kirghiz, Uyghurs, and Yakuts. The remaining sequence (AY615359) appeared in Nganasans, who speak a Samoyedic language of the Uralic language family but have mixed with Tungusic speaking and other groups in the past [60].

#### Haplogroup D

Haplogroup D, first discovered in Native Americans [47, 48, 61], is a major East Eurasian lineage, one that occurs in Northeast and Southeast Asia, and Siberia but also the Americas [15, 26, 27, 31, 32, 41, 48, 50–52, 58]. In Jetisuu Kazaks, we found three branches of haplogroup D, namely D1, D4, and D5. These lineages account for 19.5% of the maternal genetic diversity in Jetisuu Kazaks, making it the most common haplogroup in this population.

**Fig 9:**
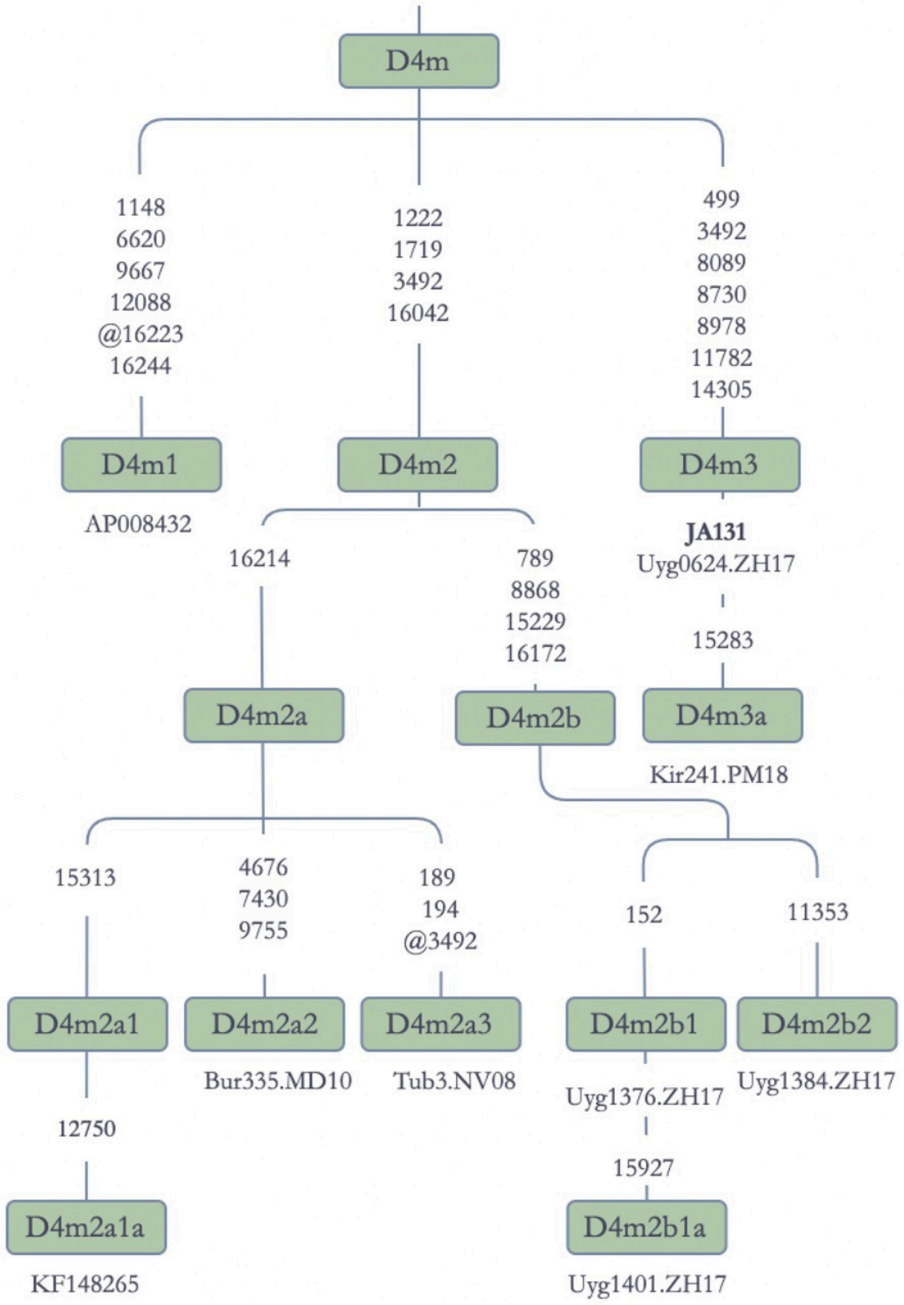
Schematic phylogenetic tree for the haplogroup D4m mitochondrial genomes. **Note:** Alt: Altaian; Bur: Buryat; Uyg: Uyghur; Kir: Kirghiz; Tib: Tibetan; Tub: Tuba/Tuva;. KF148265 (Even) and AP008432 (Japanese) were from the Phylotree Build 17.0 [46].

Within D4m (Fig 9), we observed one Jetisuu Kazak sequence. Together with close relatives in Uyghurs and Kirghiz, the Kazak sequence formed a new subbranch (D4m3). As shown in Fig 9, D4m was represented by sequences from Turkic (Kazak, Kirghiz, and Uyghur), Mongolian (Buryat) and Tungusic (Even, KF148265) speakers, along with Japanese (AP008432), implying a common Altaic origin for them.

**Fig 10:**
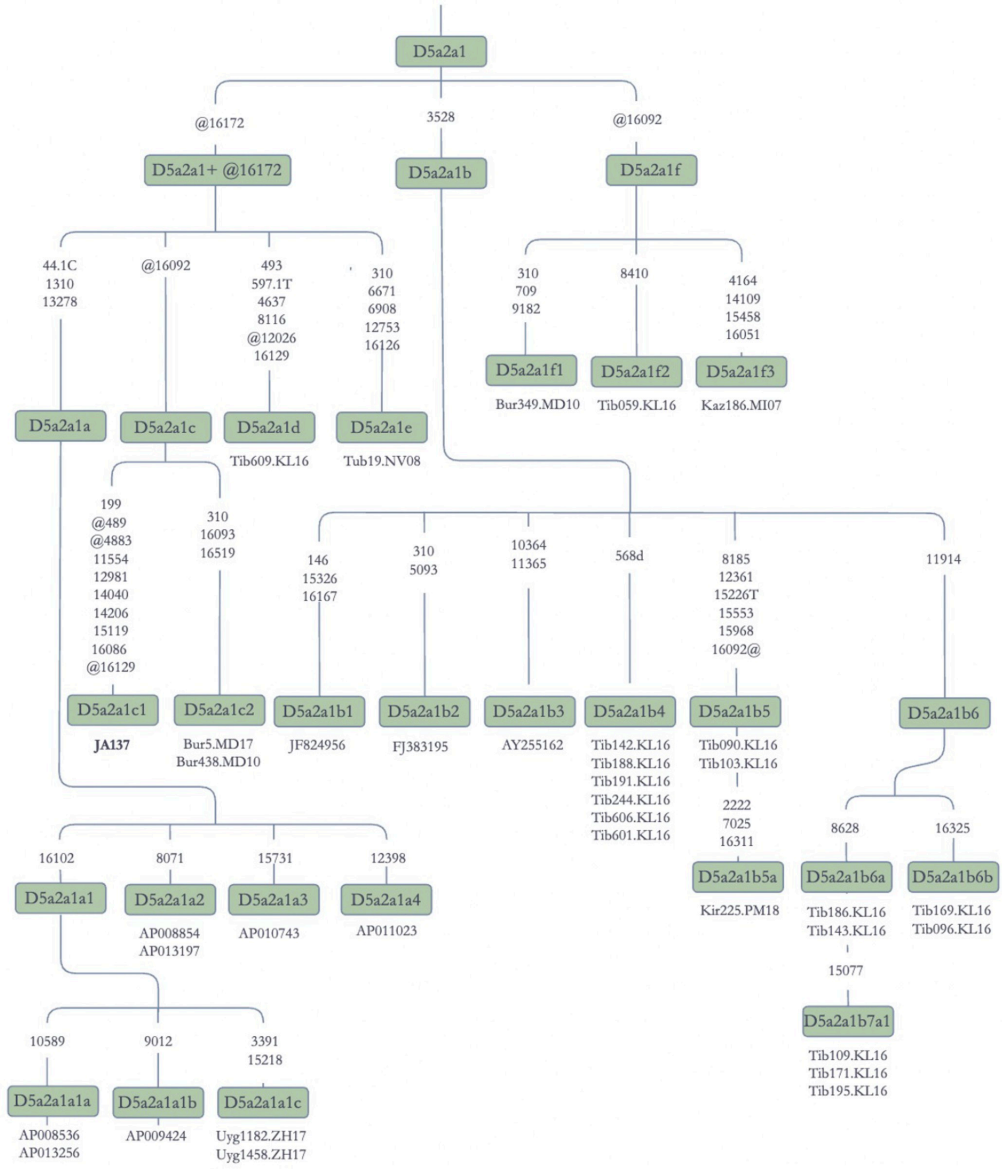
Schematic phylogenetic tree for the haplotype D5a2a1. **Note:** Bur: Buryat; Kaz: Kazak; Kir: Kirghiz; Tib: Tibetan; Uyg: Uyghur; The following sequences were obtained from the Phylotree Build 17.0 [46]: AP013256, AP008854, AP013197, AP010743, AP011023, AP008536, AP013256, and AP009424 are Japanese sequences; JF824956 is from China, but unknown ethnic origin; FJ383195 is from India; AY255162 is a Han Chinese sequence.

Most of the D5 sequences analyzed in this study belonged to D5a2a1 (Fig 10). The branching patterns of the D5 clades were complex, and the branches are formed by the sequences from Turko-Mongolian speakers, Tibetans, and Japanese.

The D5a2a1+@16172 lineage contained sequences from Buryats, Kazaks, Tibetans, Tuba, and Uyghurs. This clade had four subbranches, which included three new ones (D5a2a1c, D5a2a1d, and D5a2a1e), in addition to one (D5a2a1a) already recorded in the Phylotree Build 17.0 [46]. We also propose the new branch of D5a2a1 defined by a back mutation at 16092, but not the back mutation at 16172, which occurs in D5a2a1f (Fig 10). The newly named clade has three subbranches formed by Buryat, Kazak, and Tibetan sequences, respectively, and implying that it has a wide geographic area of distribution.

### Other haplogroups of significance

Here, we discuss the mitochondrial haplogroups F, G, H, T, and U, which are found relatively high frequencies in Jetisuu Kazaks. Haplogroups F and G are common in populations from Siberia and Central Asia [12, 15], but they occur at considerably higher frequencies in East Asia [51]. While the West Eurasian haplogroups H and T are also found in Central Asia and Siberia, their frequencies decrease in the eastward direction.

Haplogroup F is distributed in central, eastern, southern regions of Asia [12, 15, 25, 50, 51, 62, 63]. F1b1f occurs in populations that belong to Turkic (Kazak and Uyghur), Mongolic (Buryat), and Tungusic (Evenks) branches of the Altaic language family. While most subbranches of F1b1 are found in Turko-Mongolian speakers, including Buryats, Kazaks, Kirghiz, and Uyghurs, some sublineages also occur in Japanese. Similarly, F1a1 mtDNAs are found in Turko-Mongolian speakers, Japanese, and Tibetans (S4 Table), but also occur in populations in southeast Asia (i.e. Cambodia and the Orchid Island of Taiwan) [46]. Haplogroup G, which was defined by Schurr et al. [64], also reflects maternal genetic connection between Turko-Mongolian speakers and Japanese. For instance, in the clade of G2a5, Kazak and Buryat sequences are derived from a Japanese sequence (AP008897). In the same way, multiple Turko-Mongolian sequences cluster together with Japanese sequences in the clades of G2b2 and G3a2 (S1 Fig).

In our dataset, haplogroup H lineages comprised about 17% of the maternal linages in Jetisuu Kazaks, with H2 being the most common haplogroup (6.5%, Table 1). While Turkic speaking Tatars shared multiple West Eurasian haplotypes with Russians and other Slavic populations, most of the West Eurasian haplotypes found in Kazaks and other Turko-Mongolian speakers were distantly related to those occurring in the Slavic groups. In addition, some West Eurasian haplotypes exclusively occur in Turko-Mongolian speakers (e.g., H8b1). These findings strongly support the notion that the existence of West Eurasian haplotypes in modern Turko-Mongolian speakers are not simply due to Russian expansions in the recent centuries. For instance, in S4 Table, the subclade of H8b1 is comprised of Buryat, Kazak, Kirghiz, and Tuba sequences. In addition, H8b1 also includes a sequence (KF148188) from a Tungusic speaking Evenk.

**Fig 11:**
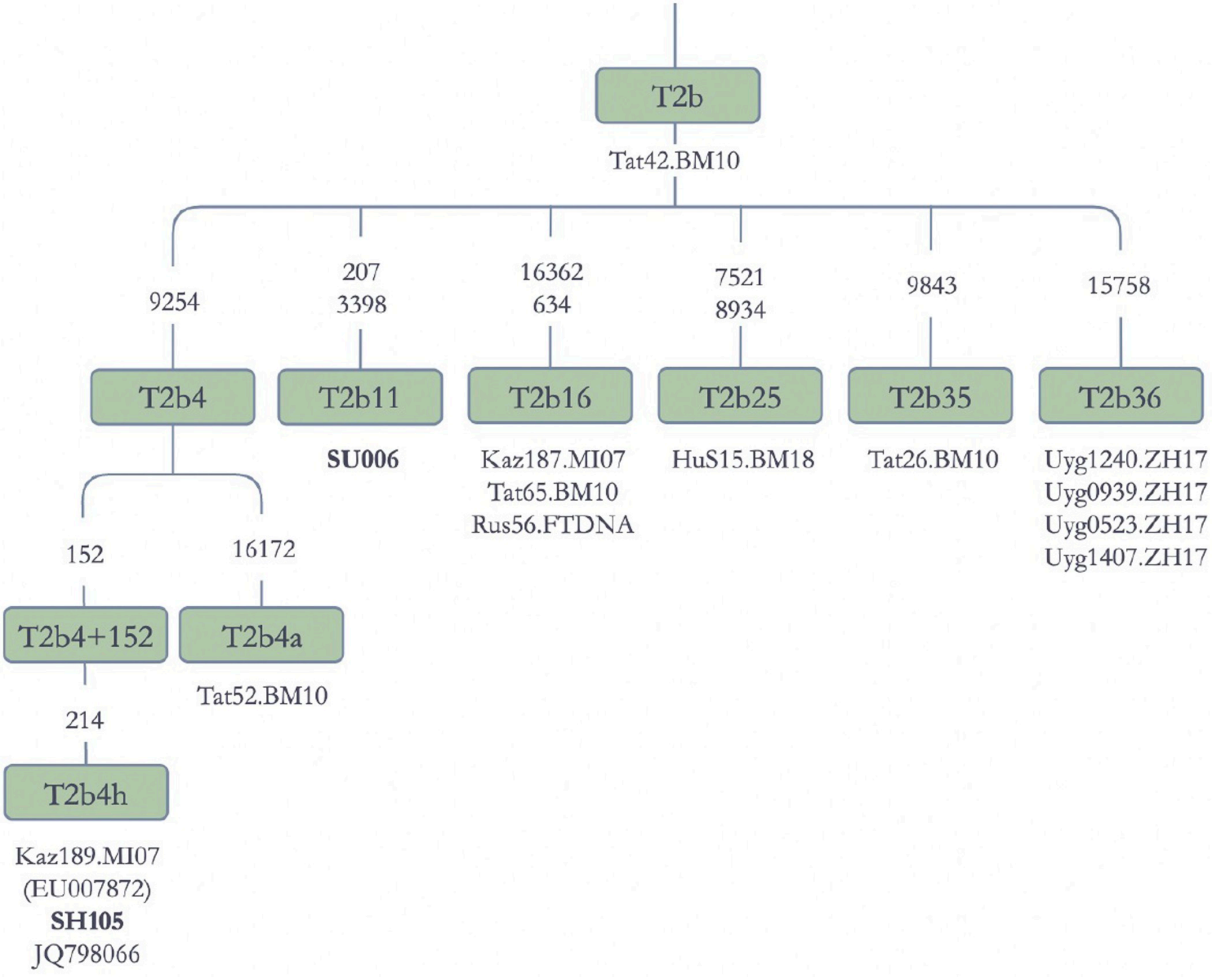
Schematic phylogenetic tree for the haplotype T2b. **Note:** Kaz: Kazak; Hu: Hungarian; Rus: Russian; Tat: Tatar; Uyg: Uyghur; **SU006** and **SH105** are Jetisuu sequences; EU007872 and JQ798066 are sequences from the Phylotree Build 17.0 [46].

It is interesting to note that, in the phylogeny of haplogroup T, Tatar sequences seemed to be ancestral to subbranches appearing in Central Asia. As shown in Fig 11, Tat42.MB10 is ancestral to T2b lineages found in Buryats, Kazaks, Kirghiz, Russians, and Hungarians. Another Tatar sequence (Tat7.BM10) is ancestral to T1a1 subbranches (S1 Text). This finding may reflect the geographic origin of haplogroup T in Central Asia and Siberia. In support of this view, T1a and T2b mtDNAs were discovered in archaeological samples of Yamna Culture sites [65] in Volga region, Russia, whence the Tatars (Tat7 and Tat42) were sampled [66].

Excluding U6 and U9, all subclades of U (U1-5 and U7-8) occurred in Turko-Mongolian speakers in Central Asia (S4 Table). While U7a was found mostly in Turko-Mongolian speakers and in the Iranian speaking Tajiks, U7b, previously believed to be absent in Central Asia [21], was discovered only in Uyghurs (S4 Table). While U4a seemed to characterize Slavic speakers, all U8 sequences in our dataset were from Turko-Mongolians, with the exception of one Hungarian sequence (S4 Table).

## Discussion

We characterized maternal lineages of Kazak populations in Jetisuu at the mtGenome level. To our knowledge, this is the first mtGenome study of human populations in Kazakhstan. Notwithstanding the small size and uniparental inheritance of mtDNA, it has provided important insights into the modern human evolution [67, 68]. The mtGenome data produced in this study will therefore be invaluable for reconstructing human population history and can serve as a reference for identification of pathogenic mutations in patients with mitochondrial disorders in Central Asians.

In previously published studies, the mitochondrial genetic profiles of Kazaks and other ethnic groups in Central Asia and Siberia were characterized through the analyses of mtDNA control region sequences and coding region SNPs [12, 14, 19, 25, 47, 61, 69, 70] and complete mtDNA sequencing [15, 20, 63, 71]. A mtDNA study of Kazaks from the Altay-Sayan region [12] revealed that they exhibit highly diverse maternal genetic profiles, harboring both West and East Eurasian maternal lineages. Our results confirmed the same pattern of maternal genetic variations in Jetisuu Kazaks, with almost all 200 individuals surveyed having distinct haplotypes (S2 and S3 Tables).

West Eurasian lineages could have become part of the maternal gene pool of Kazaks during Turko-Mongolian expansions (i.e., Turkic Khanates and Golden Horde) and/or Slavic expansions during Russian Empire and the Soviet Union, or perhaps even deeper in time, due prehistoric expansion of human populations. The comparative analyses of mtGenomes from multiple ethnic groups in Central Eurasia indicate that West Eurasian haplogroups (e.g. H, T, and U) have diverse subbranches in Kazaks and other ethnic groups in Central Asia and Siberia, with most of the subbranches being distinct from those haplotypes found in Russians and other Slavic populations. This observation indicates that Slavic expansions may not fully account for the occurrence of West Eurasian maternal lineages in Central Asia. For instance, multiple subbranches of U, including U2, U4, U5, and U7, are found in Jetisuu Kazaks, and the haplogroup U has the largest TMRCA (~ 40,000 years) among the haplogroups found in Kazaks (Table 1 and Table 2 in S1 Text). The mtDNA lineage of the 24-thousand-year-old Malta individual also belongs to basal U [72], suggesting a deep origin for this maternal lineage in Central Eurasia. Recent ancient DNA studies also found West Eurasian lineages in Iron Age populations or earlier inhabitants of the Altay Mountains [5]. Interestingly, Jetisuu Kazaks harbor the same set of West Eurasian lineages (HV, N1, J, T, U, K, W, I, and X) found in the Iron Age populations of Central Asia [5].

It can be deduced from the frequencies of these ancestral lineages in various ethnic groups in Central Asia that, the arrival of East Eurasian lineages (e.g., Haplogroups A, B, C, and D) in southern Central Asia, i.e. the Pamir Plateau, could possibly be attributed to Turko-Mongolian expansions. Examples of these expansions could include early movements of Turks and Mongols, then southward movement of Mughals, and later the expansion of the Jungarian Khanate [1]. The subbranches of haplogroups A, B, C, and D found in Uyghurs from Xinjiang and Tajiks from the Pamir are closely related to those of Altaians, Buryats, Kirghiz, and Kazaks (S4 Table). However, such a hypothesis may require further lines of evidence, given the fact that haplogroup C was found among Tarim Basin mummies [73, 74] and haplogroup D was found in an Iron Age population in Xinjiang [75]. These ancient populations predate the Turko-Mongolian expansions. In addition, haplogroup C7 occurs in northeast India [76], most likely due to prehistoric migrations.

East Eurasian haplogroups found in eastern Europe are associated with those found in Turko-Mongolians as well. A previous study tried to demonstrate paternal genetic affinities between Hungarian and Kazak populations [77], but the coalescence time of the Y-chromosome haplogroups G1 and G2 predated the historical events linking Hungarians to Central Asians. In addition, the arrival of haplogroup G1 in the Kazak steppes may date to recent historical time periods, as the age of this lineage in Kazaks was estimated to be ~ 600 years old [78]. On the other hand, it appears that maternal lineages A12a, C4a1a, and U8b1b may link Hungarians with Kazaks and other Turko-Mongolian speakers (Fig 5 and S4 Table).

In the F_ST_-based PCA analysis, Sherpa population unexpectedly exhibited a closer affinity with Turko-Mongolians than with Tibetans (Fig 4). Our analysis indicates that certain mtDNA lineages (D4j13 and D4j1a1) and some other haplotypes clearly connect Sherpas in Tibet (and Nepal) and Turko-Mongolians in Central Asia and Siberia. D4j13 occurs in Sherpas and Turkic speaking Kirghiz, Tatars, and Uyghurs, while D4j1a1 appears in Sherpas and Tibetans, as well as Mongolic speaking Buryats and Turkic speaking Kirghiz and Uyghurs (S4 Table). The similar genetic affinities can be observed between Sherpas and Turko-Mongolian speakers through haplotypes D5a2a1, C4a2, G3a, M5, M9a1a1c1, M11, M13, and the West Eurasian lineages H10, U2, and W. For instance, a Sherpa individual (Shr035.KL13) shares the basal W with a Uyghur (S4 Table). The existence of Y-chromosome haplogroups Q and R in Sherpas [79] may further suggest their connections with Turkic peoples through paternal lineages as well. However, the exact nature of the genetic affinity needs to be further evaluated by Y-chromosome and whole genome studies.

It has been noted by previous studies that Tibetans show genetic affinities with Japanese through their paternal [80] and maternal [51] lineages. In the current study, we identified several mitochondrial lineages that connect Kazaks and other ethnic groups from Central Asia with both Tibetans and Japanese. Haplogroup M10 occurs in Tibetans, but it is also found in Turkic speaking Altaians, Kazaks (e.g., AL104), and Uyghurs. Their shared maternal genetic ancestry may explain most of the maternal genetic connections between populations from the Tibetan Plateau and Turko-Mongolian groups.

This pattern of genetic diversity may have been partly shaped by recent genetic admixture. Within haplogroup A15, Uyghur sequences clustered together with those from Tibetans and Sherpas, revealing gene flow between Tibetans and Uyghurs despite their dissimilarities in language and religion. This genetic affinity could be attributed to geographic proximity, Tibetan Empire, or once flourished Buddhism in Xinjiang. An analogous genetic link was observed between Kirghiz and Tibetans in the clade of A17 (S4 Table). The directionality of gene flow, as inferred from the branching pattern, was from Tibetans into Kirghiz.

Some clades of mtDNA (D4a3b, and D4b1, D4m, and M7) present in our dataset link Japanese with Turko-Mongolian speakers. It is known that Ryukyuans from Japan harbor a high frequency of M7 mtDNAs [51]. All three subclades of M7, namely M7a, M7b, and M7c, are found in Turko-Mongolian speakers (S4 Table), including Kazaks from Jetisuu (S2 and S3 Tables). In addition, N9a mtDNAs are present in Turko-Mongolian speakers (e.g., N9a1, N9a3, N9a8, and N9a9) and occur at a considerably high frequency (2.5%) in Jetisuu Kazaks, while its sister branch N9b occurs at elevated frequencies in Ainu and Ryukyuans from Japan [51]. N9a mtDNAs are mainly found in continental Asia, including Japan [15, 31, 51, 81]. Haplogroup Y1, a subclade of N9, is also present in Buryats and Uyghurs (S4 Table), and occurs at a high frequency in Ainu people from Japan [51]. It was hypothesized that N9a might have reached East Asia through northern route after the dispersal of macrohaplogroup N from Africa [51]. Kazaks and other ethnic groups harbor several basal N and R sequences (S2–S4 Tables), and this may reflect that the basal branches of macrohaplogroup N have existed in Central Eurasia for a very long time. The discovery of a basal R mtDNA from a 45-kyr-old individual in Siberia (Ust-Ishim) [82] and a basal N mtDNA from a 34-kyr-old individual from Mongolia (Salkhit) [83] further supports the notion that some subbranches of N expanded towards East Asia through a northern route.

We found a limited number of haplogroups that exclusively characterize Turkic speakers in our dataset (e.g., A14, B4c1b2, and C4d). Interestingly, haplogroups HV1a1a, K1a17, and M13a1b1 are only found in Mongolic speaking Buryats and Barghuts. Many haplogroups, such as B4j, B4b1a3, C4a1a4, C4a2, C4b6, C5b, D4j5b, and D4j8 to name a few, are shared among Turkic and Mongolic speakers, i.e., Turko-Mongolians. Furthermore, a few other haplogroups, such as A5, B5b, D4b2b, D5a2a1, F1a1, F1b1, G2a5, G2b2, and G3a2, are found in human populations from a wide geographic area spanning from the Japanese archipelago in the East and the Kazak steppes in the West.

Some SNPs identified in certain pseudo-mtGenome sequences of Jetisuu Kazaks may potentially lead to identification of novel mtDNA lineages in haplogroup D. These may include coding region SNPs at nucleotide positions (nt) 3548 and 13830, and a control region back mutation at nt 16223 in Jetisuu Kazak mtDNA (e.g., JA005, SU009, SH012, and SH007), although we could not rule out the possibility of incorrect SNP calls on the GenoChip microarray. In addition, SNPs at nt 2396 and nt 16519 in the pseudo-mtGenomes seem to be ambiguous, as well. Therefore, the authenticity of these SNPs needs to be evaluated and confirmed by future DNA analysis. In addition to these variants, Haplogrep reported multiple back mutations for some mtGenome sequences (AL108, AL109, JA116, and SH119 etc.), suggesting that such mtGenome sequences may require additional evaluation.

Mass migration, demographic fluctuations, and genetic drift can hinder the ability to accurately pinpointing the geographic origins and TMRCA of mitochondrial lineages. Thus, to confirm our current findings regarding the TMRCA and demographic history of Jetisuu Kazak maternal lineages (S1 Text), additional analyses of the West and East Eurasian lineages found in contemporary Kazaks and other ethnic groups in Central Asia, along with an expanded analysis of ancient DNA samples, should be undertaken.

## Materials and methods

### Fieldwork and DNA preparation

Blood samples were collected from ethnic Kazak individuals during several fieldwork sessions conducted in Almati Oblast, Kazakhstan, in summer 2014 (S1 Table). All blood samples were drawn via vacutainer (BD) by medical nurses. Written informed consent was obtained from the research participants. Prior to taking bloods, all of the research participants signed an informed consent form and filled out a genealogical questionnaire. The informed consent form and questionnaire were reviewed and approved by the Ethics Committee at the Center for Life Sciences (CLS), National Laboratory Astana (NLA), Nazarbayev University (NU). After fieldwork, blood samples were stored at -86°C, and DNAs were extracted with a salt extraction method [84] with modification, or a commercial DNA extraction kit (e.g., Qiagen) at CLS, NLA, NU.

### Microarray genotyping

The genomic DNAs of 80 individuals were genotyped via the GenoChip 2.0 microarray (Illumina iSelect HD custom genotyping bead array) [85] under the framework of the of Genographic Project. The GenoChip 2.0 surveys about 750,000 SNPs from autosomes, sex chromosomes, and the mtDNA. Accordingly, in the current study, we analyzed about 3153 mitochondrial SNPs located across mitochondrial genome, i.e., from both control and coding regions of the mtDNA. Among the SNPs genotyped, about 400 of them were different from the revised Cambridge Reference Sequence (rCRS) [86], as shown in Table 2.

### Pseudo-mitochondrial genome reconstruction

Pseudo-mitochondrial genomes (pseudo-mtGenomes) were reconstructed from the SNP genotype data for 80 Kazak individuals from Jetisuu via a Python script modified from GitHub [87] (S2 Table). For the reconstruction of pseudo-mtGenomes, rCRS [86] was used as a template, and nucleotides at certain positions of rCRS were replaced with SNP calls from the GenoChip.

### Mitochondrial genome sequencing

In addition to the 80 genotyped samples, we sequenced the whole mitochondrial genomes of 120 Kazak individuals from Jetisuu. Whole mtGenome sequencing was performed on the NGS platform of Iron Torrent at the National Institute of Genomic Medicine, Mexico City, Mexico. Sequence coverages of the newly generated mtGenomes were evaluated by QUALIMAP v2.2.1 after converting the raw sequence files into BAM files [88]. The average sequence coverage of the mtGenomes was 67.5x.

### Sequence alignment

#### mtGenomes from Jetisuu

In the initial analysis, the pseudo-mtGenomes (N = 80) were aligned together with the genuine mitochondrial genomes (N = 120) using the online version of MAFFT v7.0 [89, 90]. rCRS [86] and the Reconstructed Sapiens Reference Sequence (RSRS) [91] were also included in the multiple sequence alignment (MSA). The aligned mtGenomes were further processed by the program of SNP-Sites [92] to generate an MSA VCF file. In the final stage of data analysis, BWA [93] was used for the MSA of both pseudo and genuine mtGenome sequences from Jetisuu (N = 200), for the sake of accuracy and consistency with the subsequent analyses.

#### mtGenomes from Central Eurasia

The Jetisuu Kazak mtGenome sequences (N = 200) were aligned together with 2074 mtGenomes from multiple ethnic groups across Central Eurasia (Tables 3 and S4) via BWA [93]. This step produced a SAM format alignment of 2274 mtGenome sequences. Subsequent file format conversions were performed through SAMtools [94], BAMtools [95], and BCFtools [96, 97].

#### Selection of mtGenomes for phylogenetic analysis

From the first round MSA of mtGenomes from Central Eurasia (N = 2274), subsets were selected based on haplogroup identities and their affinity with Jetisuu Kazak sequences. Thereafter, an MSA alignment was performed for each of the macro-haplogroups (A, B, C, D, F, G, H, T, and U) (Table 4), with sequences from the Phylotree Build 17.0 [46] representing major subbranches of the haplogroups also being added (Tables 4 and S5 and S6). All alignments were carried out via BWA [93], and file format conversions were performed through SAMtools [94], BAMtools [95], and BCFtools [96, 97].

### Extended Bayesian skyline plot (EBSP)

Jetisuu mtDNA sequences (N = 200) were first partitioned into HVS-I, HVS-II, and coding regions to estimate the demographic history of Jetisuu Kazaks using the EBSP method (S1 Text). The coding region was divided again based on codon start positions. Different mutation rates were used to estimate the coalescence times based on the coding and control regions of mtDNA. Two mutation rates, 31.43 x10^-8^ μ/site/year for the control region [98] and 1.71 x10^-8^ μ/site/year for the coding region [54] were used (Table 1 in S1 Text). EBSP was performed on BEAST 2.0 [99] with the Markov Chain Monte Carlo (MCMC) chain length of 30,000,000 and sampling every 3000 steps. Initial 3,000,000 iterations were considered pre-burn-in and excluded. EBSP results were reviewed with Tracer v1.7 [100] and plotted via R [101].

### Identification of mitochondrial haplogroups

Mitochondrial haplogroups were identified by analyzing either VCF or FASTA file via the online version of Haplogrep 2.0 [23]. In parallel, mitochondrial haplogroups were also obtained by analyzing FASTA format sequence files on mtPhyl 5.003 [24], which does not require MSA. No discrepancy was observed between Geno 2.0 SNP based haplogroup calls and the pseudo-mtGenome based haplogroup calls.

### PCA using mtDNA control region haplogroup frequencies

PCA analysis (Fig 2) was performed using mtDNA haplogroup frequencies for several populations in Central Asia, together with the four Kazak populations from Jetisuu. Other populations included Altaians from the Altay Republic, Russia [25]; Kazaks and Uyghurs from Kazakhstan, Kirghiz from Talas and Sari-Tash, Kyrgyzstan [13]; Mongols from Inner Mongolia, China [26]; Dungans (Hui), Mongols, Kazaks, Uyghurs, and Han Chinese from Xinjiang, China [16, 17]; Kazaks from the Altay Republic, Russia [12, 15]; Barghuts from Inner Mongolia, China [15]. Most of the comparative studies identified mtDNA haplogroups based on control region sequences, i.e., HVS-I and HVS-II, and coding region SNPs. Therefore, mtDNA haplogroup frequencies of Jetisuu Kazaks used in this PCA plot are mostly from the frequencies of basal branches of the mitochondrial phylogeny rather than terminal haplotypes. The prcomp function of base R [101] was used for the PCA analysis, and the ggplot2 [102] and factoextra packages of R [101] were used to plot PCA results.

A heatmap was created using the R packages reshape [103] and ggplot2 [102] for the haplogroup frequency data used for the PCA.

### mtGenome pairwise F_ST_ value based PCA

Pairwise F_ST_ values were calculated with pairwise_Gst_Nei function of the mmod package of R [104]. PCA was performed using the prcomp function of base R [101]. PCA plot was made by the R packages factoextra and ggplot2 [102].

### Phylogenetic analysis

Phylogenetic networks were constructed from mtGenome sequences with Network 10.1.0. [105]. Phylogenetic trees were also obtained through Haplogrep 2.0 [23] and mtPhyl 5.003 [24]. New branches and twigs, which are not available in the current version of Phylotree (v17.0) [46] and cannot be identified by Haplogrep 2.0, were reconstructed and named based on the comparative analyses of the phylogenetic trees from Haplogrep 2.0 and mtPhyl 5.003, as well as the phylogenetic networks constructed by Network v10.1.0. Schematic phylogenetic trees were drawn manually using the draw.io program [106]. mtGenome and pseudo-mtGenome sequences with ambiguous SNPs were excluded from the schematic phylogenetic trees. Jetisuu Kazak sequences were highlighted in bold. Ethnicity was used as prefix and the initials of the first author followed by the year of publication were used as suffix for the sample ID of the sequences used for comparative analysis. The mtDNA sequences from Phylotree [46] were named after their GenBank accession number, as given in the Phylotree Build 17.0 [46]. If a Phylotree sequence was also included in our initial dataset, it is shown in parenthesis in the schematic phylogenetic trees.

### Coalescence time estimation

Coalescence times (TMRCA) were estimated via the method provided by Soares and colleagues [54] with a whole mtGenome mutation rate of one mutation per 3624 years (S1 Text). Rho and sigma values were obtained on Network v10.1.0 [105].

## Supporting information

S1 Text Additional analysis of mitochondrial sequences. (PDF)

S1 Fig A phylogenetic tree of Jetisuu mitochondrial DNA (N = 200). The phylogenetic tree of the Jetisuu Kazak mtDNA was constructed via Haplogrep 2.0 [23].
(TIFF)

S1 Table Number of individuals surveyed from each of the four clans and the sampling sites. (XLSX)

S2 Table Information on the Jetisuu samples genotyped via the GenoChip microarray (N = 80). (XLSX)

S3 Table Information on the Jetisuu samples subjected to whole mitochondrial genome sequencing (N = 120). (XLSX)

S4 Table Mitochondrial genomes (N = 2074) from the Central Eurasia for comparative analyses. (XLSX)

S5 Table Mitochondrial genomes from phylotree (N = 369) for comparative analyses. (XLSX)

S6 Table Sample ID List of the mitochondrial genomes (N = 1155) used for the second round mtGenome analysis. Ethnicity and GenBank ID information of the samples can be found in S2–S4 Tables.
(XLSX)

S7 Table Sample ID list of mitochondrial genomes from the Central Eurasia (N = 1954) and Jetisuu (N = 120, excluding pseudo-mtGenomes) that were used for the mtGenome F_ST_ value based PCA analysis. Ethnicity and GenBank ID information of the samples can be found in S3 and S4 Tables.
(XLSX)
