# Supplementary material for: Kazak mitochondrial genomes provide insights into the human population history of Central Eurasia
Source: PLoS One. 2022 Nov 29;17(11):e0277771. doi: 10.1371/journal.pone.0277771 (PMC9707748; doi:10.1371/journal.pone.0277771)
Supplement: S1 Text — (PDF) [file pone.0277771.s001.pdf]

Kazak mitochondrial genomes provide insight into the human population history in Central Eurasia

Askapuli A[1,2,3,11*], Vilar MG[9,13], Garcia-Ortiz H[4], Zhabagin M[1,2,3], Sabitov Z[5], Akilzhanova A[3], Ramanculov E[1,2], Schamiloglu U[1], Martínez-Hernández A[4], Contreras-Cubas C[4], Barajas-Olmos F[4], Schurr TG[8], Zhumadilov Z[3,14], Flores-Huacuja M[4], Orozco L[4], Hawks J[11,12], and Saitou N[6,7,10]

[1]School of Sciences and Humanities, Nazarbayev University, Nur-Sultan, Kazakhstan
[2]National Center for Biotechnology, Nur-Sultan, Kazakhstan
[3]National Laboratory Astana, Nazarbayev University, Nur-Sultan, Kazakhstan
[4]Immunogenomics and Metabolic Diseases Laboratory, National Institute of Genomic Medicine, Mexico City, Mexico
[5]L.N. Gumilyov Eurasian National University, Nur-Sultan, Kazakhstan
[6] Population Genetics Laboratory, National Institute of Genetics, Mishima, Shizuoka, Japan
[7]Department of Biological Sciences, Graduate School of Science, University of Tokyo, Tokyo, Japan
[8]Department of Anthropology, University of Pennsylvania, Philadelphia, Pennsylvania, United States of America
[9]The Genographic Project, National Geographic Society, Washington DC, United States of America
[10]Advanced Medical Research Center, Faculty of Medicine, University of the Ryukyus, Okinawa Ken, Japan
[11]Department of Integrated Biology, University of Wisconsin-Madison, Wisconsin, United States of America
[12]Department of Anthropology, University of Wisconsin-Madison, Wisconsin, United States of America
[13]Department of Anthropology, University of Maryland, College Park, Maryland, United States of America
[14]School of Medicine, Nazarbayev University, Nur-Sultan, Kazakhstan

* Corresponding author
E-mail: ayken.askapuli@wisc.edu

Supplementary Text 1. Additional Analysis of Mitochondrial Sequences

Estimation of Demographic History

For the estimation of demographic history via the Extended Bayesian Skyline Plot (EBSP) method of BEAST [1–3], mitochondrial genomes were first partitioned into HVS-I, HVS-II, and coding region. Thereafter, the coding region was divided again based on codon start positions. Different mutation rates were used for the coding and control regions of mtDNA. For the control region (i.e., HVS), 31.43 $\times 10^{-8}$ μ/site/year and for the coding region, 1.71 $\times 10^{-8}$ μ/site/year were used (Table 1).

Table 1. Partitioning of Mitochondrial Genomes and the Mutation Rates used for EBSP.

Subset	Partition Names	Charset	Nr. of Sites	Best Model	Mutation Rate (**μ**/site/year)
1	HVS-II	1-576	1122	GTR+I+G	31.43 $\times 10^{-8}$ [1]
	HVS-I	16024-16569			
2	CodingR_pos1	577-16023\3	15447	TRN+I+G	1.71 $\times 10^{-8}$ [2]
	CodingR_pos2	578-16023\3			
	CodingR_pos3	579-16023\3			

[1] The mutation rate is from Rieux et al. 2014 [4];
[2] The mutation rate is from Soares et al. 2009 [5];

As shown in EBSP (Figure 1), there was a sharp increase in effective population size of the ancestors of Jetisuu Kazaks about 25 kya. This date predates the Last Glacial Maximum (LGM), implying that the ancestral population(s) was not affected by glaciation in the northern

Figure 1. Extended Bayesian Skyline Plot for the mtGenomes from Jetisuu

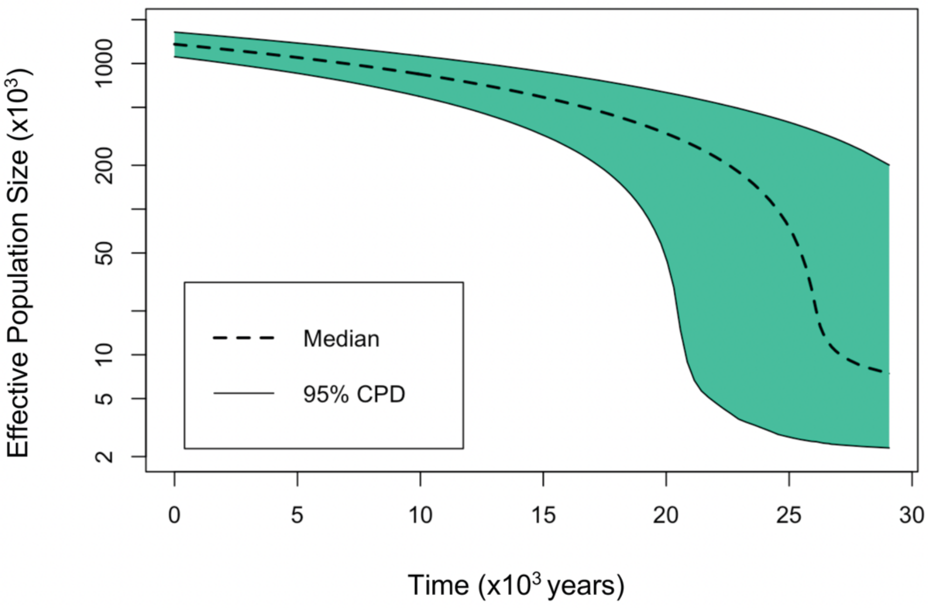

hemisphere and might have experienced population growth due to rich natural resources for subsistence. However, this may hold true under the assumptions: (1) Maternal gene pool of Kazaks was formed 25 kya and maternal lineages remained unchanged until now; (2) Mutation rates of mtDNA used in EBSP are reliable; and (3) the mitochondrial sequences under investigation have never been subject to selection. Our data may violate the assumption (1). It is evident from mitochondrial haplogroups found in Jetisuu Kazaks (Table 1) that although East Eurasian lineages comprised the core maternal gene pool of Kazaks, West Eurasian lineages might have been introduced to the population relatively recently. Age estimations of individual haplogroups are relatively young (Table 2) compared to the time period of population expansion estimated by EBSP.

Coalescence Time Estimation

As given in Table 2, the estimated TMRCA (Time to the Most Recent Common Ancestor) of haplogroup C was 55795 years, that is the oldest haplogroup in Kazaks. However, the estimated TMRCA was obtained by analyzing 15 haplogroup C sequences, including a single C7 sequence. The presence of C7 in Kazaks must have resulted from a recent admixture. Both C4 and C5 are about 20,000 years old, while their combined TMRCA is about 29,000 years (Table 2).

Table 2. Coalescence Time Estimation for the Major mtDNA Lineages from Jetisuu.

Hg		N	Rho	Sigma	TMRCA (years)	Lower 95% CI	Upper 95% CI
A		8	7.25	1.1726	19,729	13,236	26,423
B							
	B5	3	8.3333	1.7951	22,860	12,856	33,322
C		15	19	3.3353	55,795	35,182	77,525
	C4'5	14	10.357	1.6645	28,824	19,283	38,736
	C4	9	8	1.5316	21,892	13,365	30,756
	C5	5	7.2	1.5492	19,585	11,059	28,465
D		28	11.786	1.8038	33,122	22,619	44,041
	D4	26	8.8462	0.85658	24,358	19,492	29,326
F							
	F1	11	11.273	1.8675	31,571	20,772	42,824
G		14	14.071	2.3744	40,135	26,036	54,903
	G2	13	12.846	2.1248	36,355	23,875	49,392
H		27	5.7778	0.58678	15,546	12,338	18,806
HV		8	7.75	1.25	21,168	14,207	28,356
	HV2	3	5	1.7321	13,370	4,172	23,039
N9							
	N9a	5	7.8	1.456	21,313	13,221	29,711
T		13	10.077	1.339	27,990	20,311	35,912
	T2	11	8.6364	1.3016	23,744	16,412	31,314
U		15	14.533	1.4514	41,573	32,826	50,565
	U4	3	15.667	2.708	45,129	28,831	62,253
	U5	6	10.833	2.0207	30,248	18,648	42,386

The haplogroups with the largest TMRCAs include F1 (~31K), G2 (~36K), U4 (~45K), and U5 (~30K). These haplogroups seem to be older than haplogroups A, B5, C4, C5, and D4, which constitute the core of maternal gene pool of Kazaks. West Eurasian lineages T2, H, and HV are about 23K, 15K, and 21K years old, respectively.

Additional Lineages of Haplogroups C, D, and T

C4a2 has three branches, and two of them, C4a2a and C4a2c, have representatives from Jetisuu Kazaks (Figure 2). C4a2a is comprised of three newly named subbranches, C4a2a1, C4a2a2, and C4a2a3. C4a2a3 is represented by a single sequence (JA143), while C4a2a1 has multiple sub-branches represented by Altaian, Buryat, Kazak, and Uyghur sequences, clearly suggesting C4a2a as one of the maternal founder lineages of Turko-Mongolian speakers. The sub-branches of C4a2a1 were named as C4a2a1a through C4a2a1h.

Figure 2. Schematic phylogenetic tree for the haplogroup C4a2 mitochondrial genomes.
Note: Alt: Altaian; Bur: Buryat; Uyg: Uyghur; Kir: Kirghiz; Taj: Tajik;

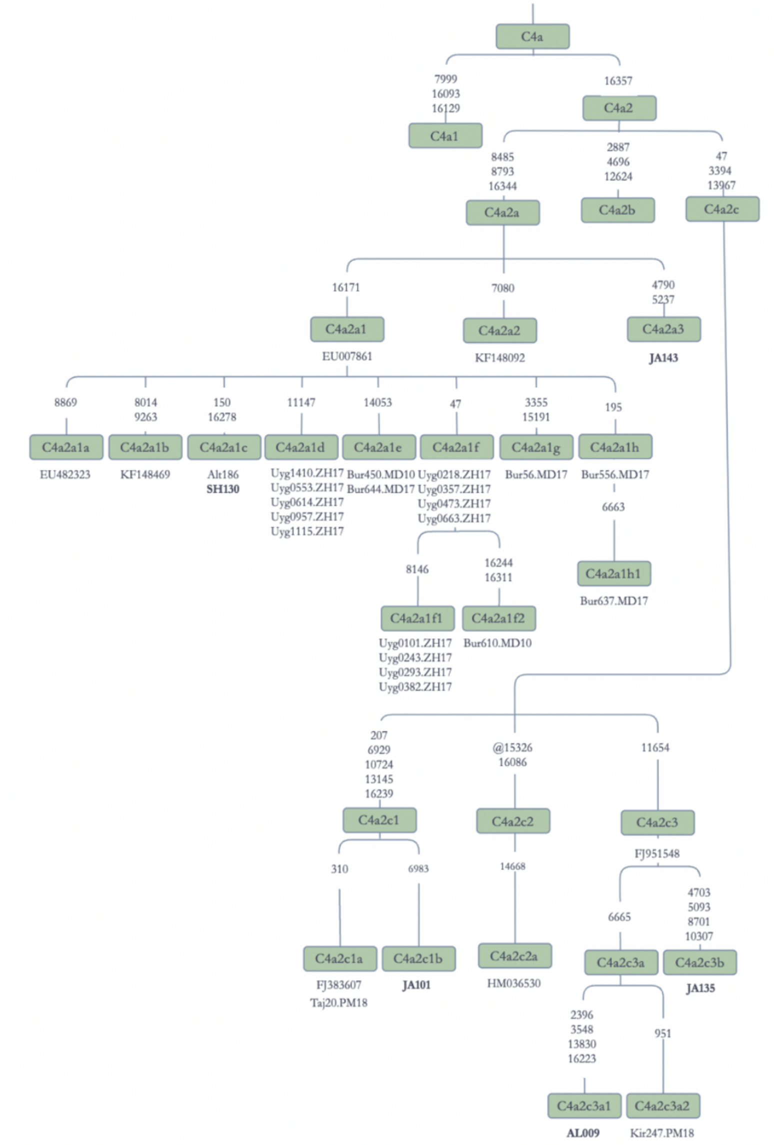

The Jetisuu Kazak sequence SH130 formed a subclade (C4a2a1c) together with an Altaian sequence. As shown in Figure 2, under C4a2c1, one Kazak sequence (JA101) clustered together with sequences from a Tajik sequence and a sequence from India (FJ383607, Jenu Kuruba [6]). Within C4a2c3, there were two Kazak sequences, AL009 and JA135, which were named C4a2c3a and C4a2c3b, respectively. The C4a2c3a Kazak sequence has a sister sequence in Kirghiz (Figure 2). A Barghut sequence (FJ951548) formed the basal branch of C4a2c3, implying an eastern origin for the haplotype. C4a2c2, another branch of C4a2c, is represented by a Ladakh sequence (HM036530) from India [7]. The tree topology as given in Figure 2, may imply that C4a2 originated in a Turko-Mongolian stock population in Central Asia or Siberia. Besides, the presence of C4a2c2 in India could be viewed as genetic legacy of the steppe nomads from Central Asia.

Figure 3. Schematic phylogenetic tree for the haplogroup C4b mitochondrial genomes.

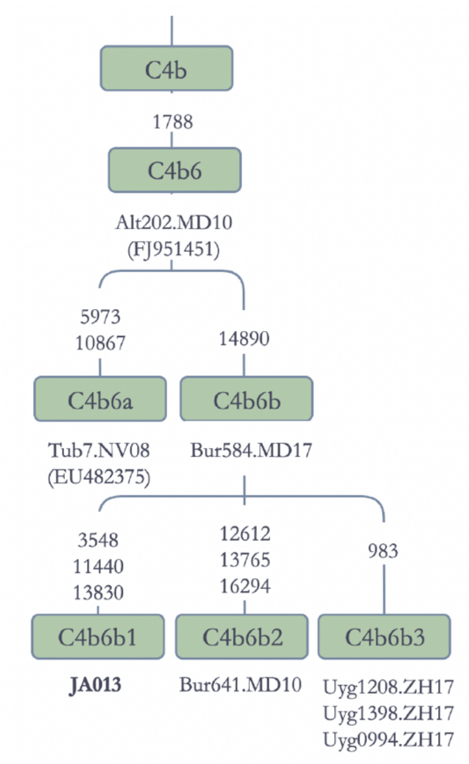

Figure 4. Schematic phylogenetic tree for the haplogroup C4d mitochondrial genomes.

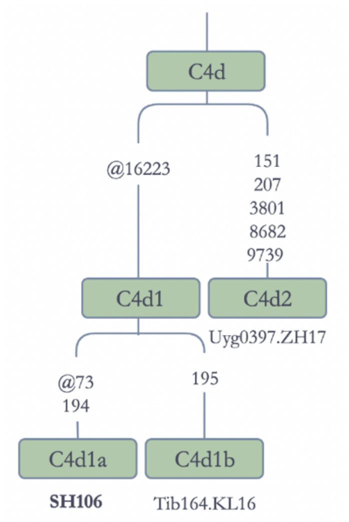

Note: Bur: Buryat; Uyg: Uyghur; Tib: Tibetan; Tub: Tuba;

A single Jetisuu Kazak sequence (JA013) belonged to the subbranch of C4b6 (Figure 3). C4b6 forms a single branch represented by two sequences (Alt202 and Tuba7) in the current version of Phylotree (v17.0). mtDNA sequences included in our dataset have produced additional twigs of the branch, as given in Figure 3. The branch formed by the Kazak sequence (JA013)

from Jetisuu was named as C4b6b1. C4d was comprised of three sequences, one each in a Kazak, Tibetan and Uyghur. The branch formed by the Kazak sequence (SH106) was named C4d1a (Figure 4). The genetic link between Kazaks and Tibetans could be made possible through either Kirghiz or Uyghurs.

Haplogroup C7 (Figure 5) has one sequence (SH132) from Jetisuu Kazaks, which was named C7a1c2a. All C7 Uyghur sequences also belonged to C7a1c. C7a1 has sub-branches (FJ383630, FJ383627, and FJ383636 in Figure 5) in India [6].

Figure 5. Schematic phylogenetic tree for the haplogroup C7 mitochondrial genomes.

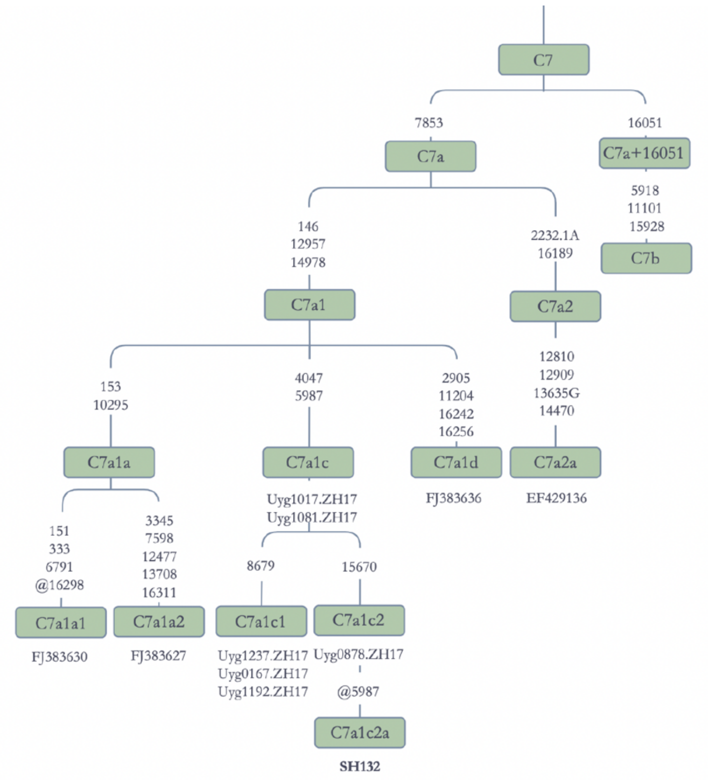

Note: Uyg: Uyghur;

Figure 6. Schematic phylogenetic tree for the haplogroup D4a.

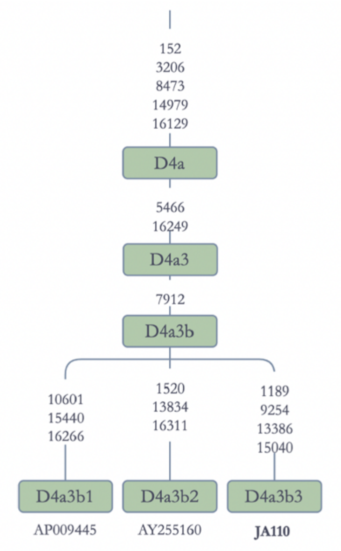

Note: AP009445 (Japanese) and AY255160 (Han Chinese) are from the Phylotree Build 17.0.

As shown in Figure 6, D4a3b had three subbranches, one represented by a Japanese (AP009445), another by a Han Chinese (AY255160), and a third by a Jetisuu Kazak. Thus, D4a3b has a wide geographic distribution. The Jetisuu Kazak sequence, JA110, was named D4a3b3.

Subbranch of D4b, D4b2b, has sequences in multiple ethnic groups including Altaians, Buryats, Kazaks, Tatars, Tubas (Tuvans), Uyghurs, and Japanese (e.g. AP008264) (Figure 7). Thus, D4b2b could be considered as a founder haplotype for Altaic speakers.

Figure 7. Schematic phylogenetic tree for the haplotype D4b2b.

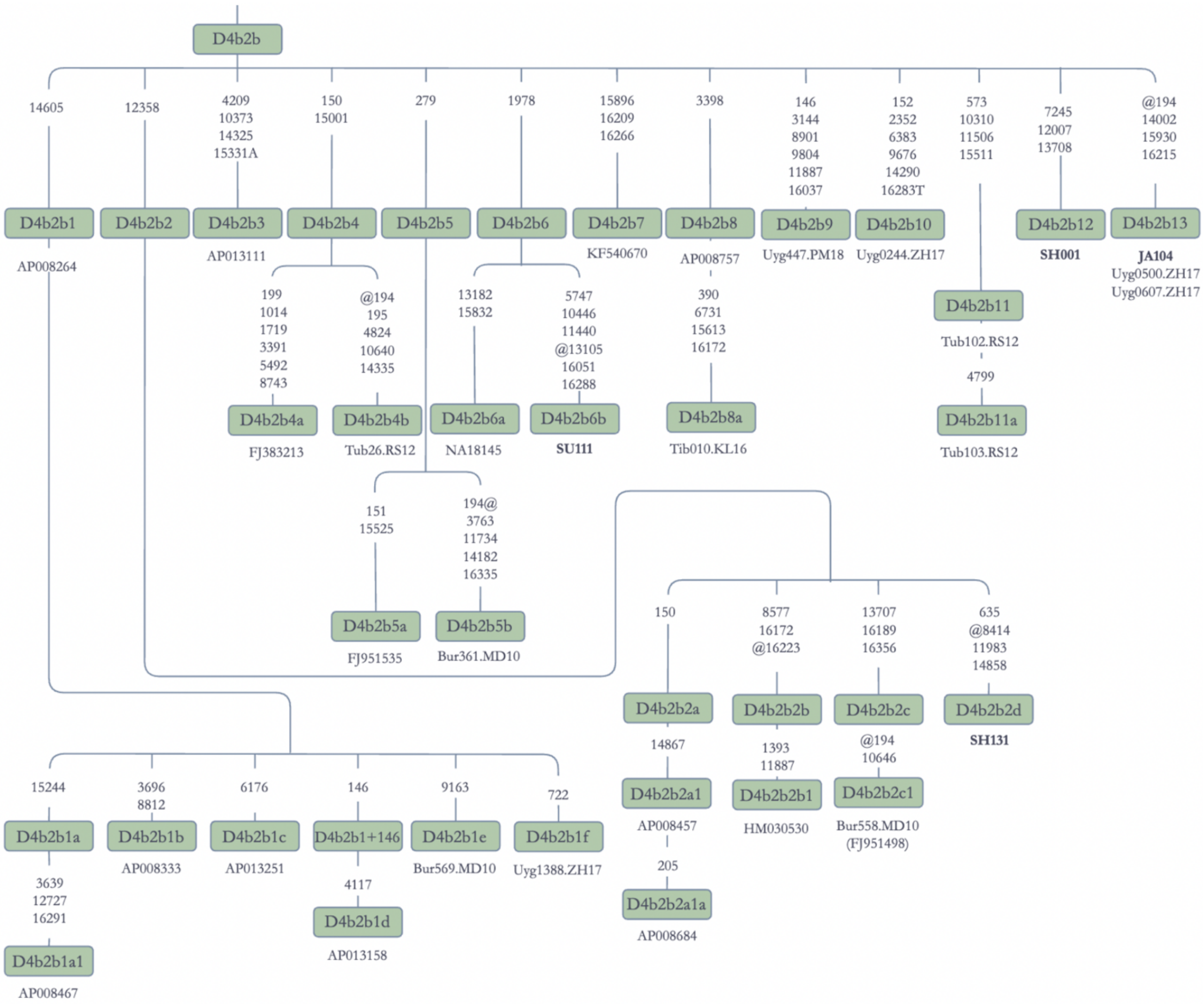

Note: Bur: Buryat; Uyg: Uyghur; Kir: Kirghiz; Tib: Tibetan; Tub: Tuba/Tuva;

As shown in Figure 8, two Kazak sequences belonged to D4j, one from the current study, and one from a previously published study [8]. The clade is diverse, with D4j5 and D4j8 being present in Buryat, Kirghiz, and Uyghur sequences, along with those of Kazaks, suggesting a common Turko-Mongolian origin. Interesting, D4j5a was represented by a Yukaghir sequence (EU482325). Yukaghir language is believed to belong to the Uralic language family, or at least, distantly related to the Uralic language family.

As shown in Figure 9, another Tatar sequence (Tat7.BM10) is ancestral to T1a1 subbranches. This may reflect the geographic origin of haplogroup T in Central Asia and Siberia. T1a and T2b were discovered in archaeological samples of Yamna Culture sites [9] in Volga region, Russia, whence the Tatars were sampled [10].

Figure 8. Schematic phylogenetic tree for the haplogroup D4j mitochondrial genomes.

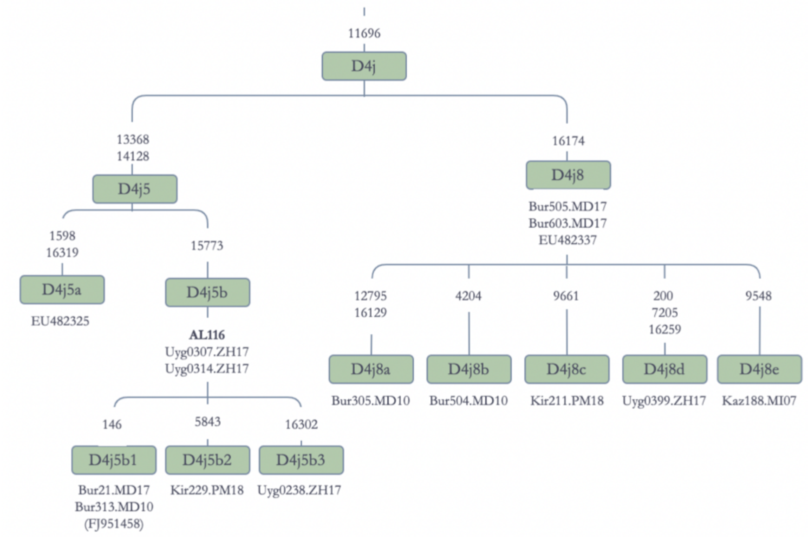

Note: Bur: Buryat; Kaz: Kazak; Kir: Kirghiz; Uyg: Uyghur; EU482325 (Yukaghir), FJ951458 (Buryat) and EU482337 (Buryat) were from the Phylotree Build 17.0;

Figure 9. Schematic phylogenetic tree for the haplotype T1a1.

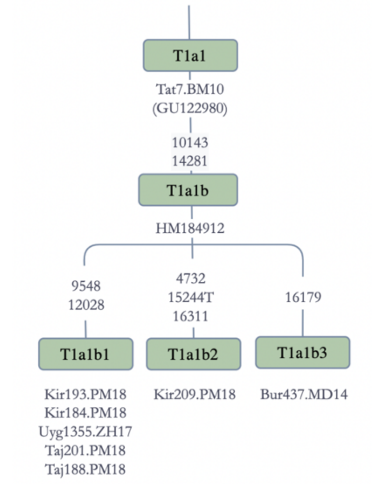

Note: Bur: Buryat; Kir: Kirghiz; Taj: Tajik; Tat: Tatar; Uyg: Uyghur; HM184912 is a Czech sequence from the Phylotree Build 17.0;

References

1. Drummond AJ, Rambaut A. BEAST: Bayesian evolutionary analysis by sampling trees. BMC Evolutionary Biology. 2007;7. doi:10.1186/1471-2148-7-214
2. Bouckaert R, Heled J, Kühnert D, Vaughan T, Wu C-H, Xie D, et al. BEAST 2: A software platform for Bayesian evolutionary analysis. PLoS Computational Biology. 2014;10: e1003537: 1-6. doi:10.1371/JOURNAL.PCBI.1003537
3. Bouckaert R, Vaughan TG, Barido-Sottani J, Duchêne S, Fourment M, Gavryushkina A, et al. BEAST 2.5: An advanced software platform for Bayesian evolutionary analysis. PLoS Computational Biology. 2019;15. doi:10.1371/journal.pcbi.1006650
4. Rieux A, Eriksson A, Li M, Sobkowiak B, Weinert LA, Warmuth V, et al. Improved calibration of the human mitochondrial clock using ancient genomes. Molecular Biology and Evolution. 2014;31: 2780. doi:10.1093/MOLBEV/MSU222
5. Soares P, Ermini L, Thomson N, Mormina M, Rito T, Röhl A, et al. Correcting for purifying selection: an improved human mitochondrial molecular clock. Am J Hum Genet. 2009;84: 740–59. doi:10.1016/j.ajhg.2009.05.001
6. Chandrasekar A, Kumar S, Sreenath J, Sarkar BN. Updating Phylogeny of Mitochondrial DNA Macrohaplogroup M in India : Dispersal of Modern Human in South Asian Corridor. October. 2009;4. doi:10.1371/journal.pone.0007447
7. Sharma V, Singh L, Thangaraj K, Nandan A, Sharma VK. Phylogenomic study of a concealed Ladakh tribe of the Great Himalayas. pp. GenBank: HM036576.036571. 2010.
8. Ingman M, Gyllensten U. Rate variation between mitochondrial domains and adaptive evolution in humans. Human Molecular Genetics. 2007;16: 2281–2287. doi:10.1093/hmg/ddm180
9. Wilde S, Timpson A, Kirsanow K, Kaiser E, Kayser M, Unterländer M, et al. Direct evidence for positive selection of skin, hair, and eye pigmentation in Europeans during the last 5,000 y. Proc Natl Acad Sci U S A. 2014;111: 4832–4837. doi:10.1073/pnas.1316513111
10. Malyarchuk B, Derenko M, Denisova G, Kravtsova O. Mitogenomic diversity in Tatars from the Volga-Ural region of Russia. Molecular Biology and Evolution. 2010;27: 2220–2226. doi:10.1093/molbev/msq065
